# Supplementary material for: Senescence-directed nanotherapy ameliorates fibrosis and overcomes immune exclusion in cancer
Source: Science. Author manuscript; Available in PMC 2026 Oct 5. (PMC13637827; doi:10.1126/science.aeg4791)
Supplement: Supplementary file 1 [file NIHMS2207444-supplement-Supplementary_Material_with_Figure_S1-S14__Figure_S1-S14_legends__and_Table_S1-S6_description.pdf]

Supplementary Materials for

Senescence-directed nanotherapy ameliorates fibrosis and overcomes immune exclusion in cancer

Clemens Hinterleitner[1]†, Valentin J. A. Barthet[1]†, Hailey V. Goldberg[1,2]†, Kristen C. Vogt[3,4]‡, Ana Marie Perea[3,5]‡, Logan R. Hillger[3]‡, Stephen Ruiz[3,2]‡, Domhnall McHugh[1], Yu-Jui Ho[1], Almudena Chaves-Perez[1], Maria Skamagki[6], Sara Flowers[7], Hannah C. Styers[1,5], Natasha Rekhtman[8], Xueqian Zhuang[1], Gabriel Dessotti Barretto[9], Xiang Li[1], Jadae T. Watson[1], Wei Luan[1], Janelle Simon[1], Tuomas Tammela[1], Rui Gardner[9], Charles M. Rudin[10,2], Paul B. Romesser[7,11], Matthew J. Bott[6], Aveline Filliol[1]*, Daniel A. Heller[3,2]*, Scott W. Lowe[1,12]*

Corresponding authors: lowes@mskcc.org, hellerd@mskcc.org, and filliola@mskcc.org

This supplementary material includes:

Figs. S1 to S14
Tables S1 to S6 description

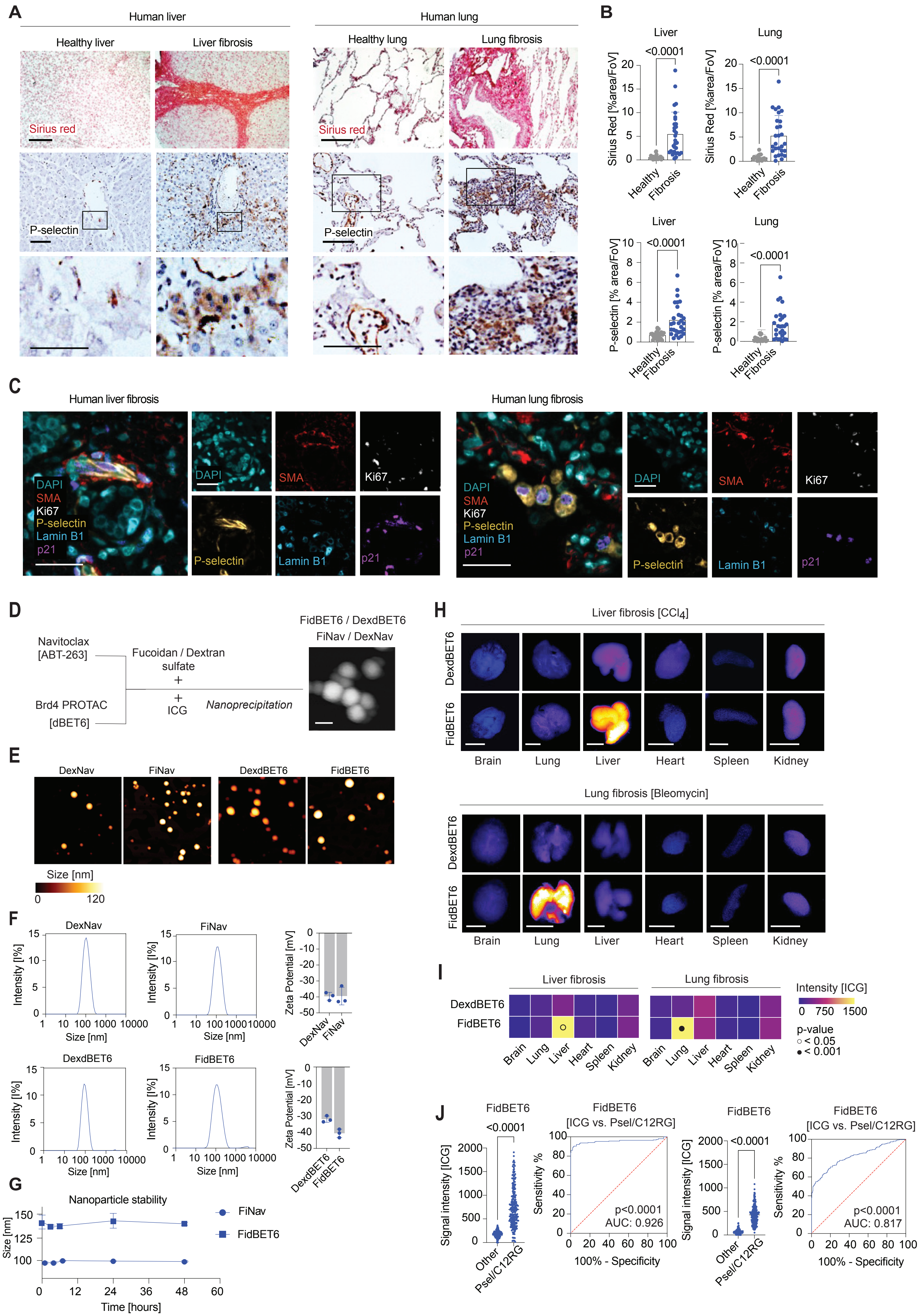
A
Human liver
Healthy liver
Liver fibrosis
Sirius red
P-selectin
Human lung
Healthy lung
Lung fibrosis
Sirius red
P-selectin
B
Liver
Lung
<0.0001
Sirius Red [%area/FoV]
P-selectin [% area/FoV]
Healthy
Fibrosis
C
Human liver fibrosis
Human lung fibrosis
DAPI
SMA
Ki67
P-selectin
Lamin B1
p21
D
Navitoclax [ABT-263]
Brd4 PROTAC [dBET6]
Fucoidan / Dextran sulfate
+
+
ICG
Nanoprecipitation
FidBET6 / DexdBET6
FiNav / DexNav
E
DexNav
FiNav
DexdBET6
FidBET6
Size [nm]
0
120
F
DexNav
FiNav
DexdBET6
FidBET6
Intensity [%]
Size [nm]
Zeta Potential [mV]
G
Nanoparticle stability
FiNav
FidBET6
Size [nm]
Time [hours]
H
Liver fibrosis [CCl4]
Lung fibrosis [Bleomycin]
DexdBET6
FidBET6
Brain
Lung
Liver
Heart
Spleen
Kidney
I
Liver fibrosis
Lung fibrosis
DexdBET6
FidBET6
Brain
Lung
Liver
Heart
Spleen
Kidney
Intensity [ICG]
0
750
1500
p-value
○ < 0.05
● < 0.001
J
FidBET6
<0.0001
Signal intensity [ICG]
Other
Psel/C12RG
FidBET6
[ICG vs. Psel/C12RG]
Sensitivity %
100% - Specificity
p<0.0001
AUC: 0.926
p<0.0001
AUC: 0.817

Figure S1: Expression and SMNP-mediated targeting of P-selectin-expressing cells in fibrotic tissues. (Related to Figure 1)

(**A-B**) Representative images (A) and quantification (B) of collagen deposition (Sirius Red) and P-selectin expression (IHC) in human fibrotic and cirrhotic liver (healthy, n=21; fibrotic, n=30 cases) and lung (healthy, n=2; fibrotic, n=28 cases). Scale bars, 100 μm and 25 μm (magnification). (**C**) Representative images of P-selectin^{+} senescent cells, defined as p21^{+}, Ki67^{-}, LaminB1^{low} in human liver fibrosis (left) and lung fibrosis (right). Scale bars, 20 μm. (**D**) Schematic of drug (Navitoclax or dBET6) nanoprecipitation into sulfated fucoidan (Fi) or dextran (Dex) polysaccharides co-formulated with indocyanine green (ICG). (**E**) Representative atomic force microscopy images used to assess nanoparticle size (DexNav, FiNav, DexdBET6, FidBET6). (**F**) Dynamic light scattering (DLS) size distribution from representative individual nanoprecipitations of DexNav, FiNav, DexdBET6, and FidBET6 nanoparticles (left, middle) and surface zeta potential confirming dextran and fucoidan surface functionalization (right). (n=3 nanoprecipitation samples, 1 experiment). (**G**) FiNav and FidBET6 nanoparticle stability assay in saline. (**H-I**) Representative macroscopic images (H) and quantification (I) of ICG^{+} nanoparticle enrichment (FidBET6 and DexdBET6) across organs in murine models of liver and lung fibrosis at 12 h post nanoparticle injection; (3 mice/group). Scale bars, 500 μm. (**J**) ICG signal intensity in P-selectin^{+} senescent cells (defined as C12RG^{+}; n=3 mice per group, left) and ROC curve of ICG specificity for P-selectin^{+} senescent cells (n=3 mice per group, right). Each dot represents one patient (B), one independent nanoparticle batch generated (F), or one cell (J). Data are presented as mean ± s.e.m. Mann-Whitney test (B,C,J), one-way ANOVA with Sidak's multiple comparisons test (I), and receiver operating characteristic (ROC) analysis (J).

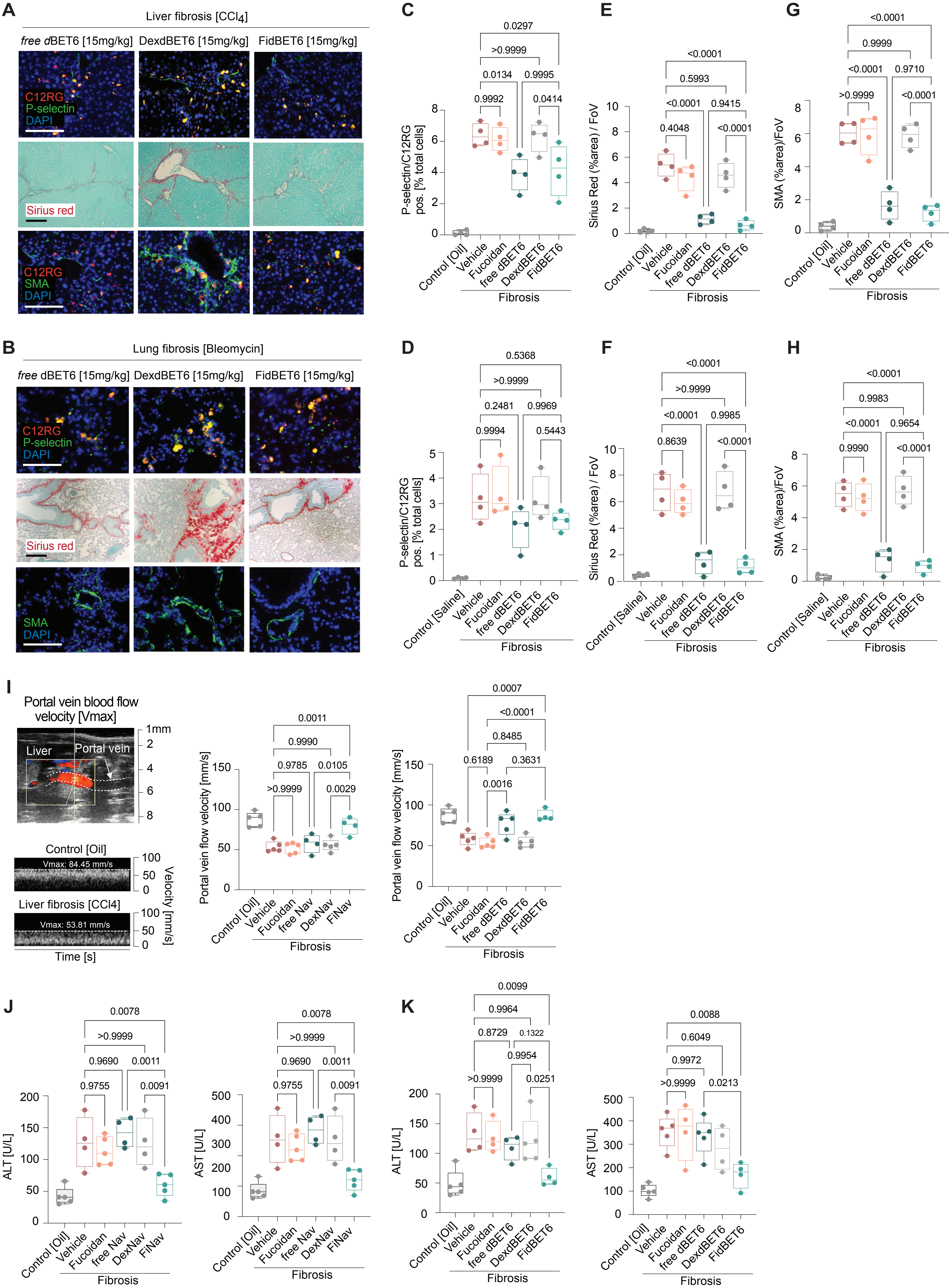

A
Liver fibrosis [CCl4]
free dBET6 [15mg/kg]
DexdBET6 [15mg/kg]
FidBET6 [15mg/kg]
C12RG
P-selectin
DAPI
Sirius red
C12RG
SMA
DAPI
B
Lung fibrosis [Bleomycin]
free dBET6 [15mg/kg]
DexdBET6 [15mg/kg]
FidBET6 [15mg/kg]
C12RG
P-selectin
DAPI
Sirius red
SMA
DAPI
C
P-selectin/C12RG pos. [% total cells]
0.0297
>0.9999
0.0134
0.9995
0.9992
0.0414
Control [Oil]
Vehicle
Fucoidan
free dBET6
DexdBET6
FidBET6
Fibrosis
E
Sirius Red (%area) / FoV
<0.0001
0.5993
<0.0001
0.9415
0.4048
<0.0001
Control [Oil]
Vehicle
Fucoidan
free dBET6
DexdBET6
FidBET6
Fibrosis
G
SMA (%area)/FoV
<0.0001
0.9999
<0.0001
0.9710
>0.9999
<0.0001
Control [Oil]
Vehicle
Fucoidan
free dBET6
DexdBET6
FidBET6
Fibrosis
D
P-selectin/C12RG pos. [% total cells]
0.5368
>0.9999
0.2481
0.9969
0.9994
0.5443
Control [Saline]
Vehicle
Fucoidan
free dBET6
DexdBET6
FidBET6
Fibrosis
F
Sirius Red (%area) / FoV
<0.0001
>0.9999
<0.0001
0.9985
0.8639
<0.0001
Control [Saline]
Vehicle
Fucoidan
free dBET6
DexdBET6
FidBET6
Fibrosis
H
SMA (%area)/FoV
<0.0001
0.9983
<0.0001
0.9654
0.9990
<0.0001
Control [Saline]
Vehicle
Fucoidan
free dBET6
DexdBET6
FidBET6
Fibrosis
I
Portal vein blood flow velocity [Vmax]
Liver
Portal vein
1mm
Control [Oil]
Vmax: 84.45 mm/s
Liver fibrosis [CCl4]
Vmax: 53.81 mm/s
Velocity [mm/s]
Time [s]
Portal vein flow velocity [mm/s]
0.0011
0.9990
0.9785
0.0105
>0.9999
0.0029
Control [Oil]
Vehicle
Fucoidan
free Nav
DexNav
FiNav
Fibrosis
Portal vein flow velocity [mm/s]
0.0007
<0.0001
0.8485
0.6189
0.3631
0.0016
Control [Oil]
Vehicle
Fucoidan
free dBET6
DexdBET6
FidBET6
Fibrosis
J
ALT [U/L]
0.0078
>0.9999
0.9690
0.0011
0.9755
0.0091
Control [Oil]
Vehicle
Fucoidan
free Nav
DexNav
FiNav
Fibrosis
AST [U/L]
0.0078
>0.9999
0.9690
0.0011
0.9755
0.0091
Control [Oil]
Vehicle
Fucoidan
free Nav
DexNav
FiNav
Fibrosis
K
ALT [U/L]
0.0099
0.9964
0.8729
0.1322
0.9954
>0.9999
0.0251
Control [Oil]
Vehicle
Fucoidan
free dBET6
DexdBET6
FidBET6
Fibrosis
AST [U/L]
0.0088
0.6049
0.9972
>0.9999
0.0213
Control [Oil]
Vehicle
Fucoidan
free dBET6
DexdBET6
FidBET6
Fibrosis

Figure S2: SMNPs improve fibrosis and organ function. (Related to Figure 2)
(**A**-**H**) Analysis of collagen deposition (Sirius Red), fibroblast activation (SMA), and P-selectin$^+$ senescent cells in CCl_4-induced fibrotic livers (A) or bleomycin-induced fibrotic lungs (B) treated with free dBET6, Dex-dBET6 nanoparticles, or FidBET6 nanoparticles (A-B) and quantification (4 mice/group) including control groups (C-H). Scale bars, 150 µm. (**I**) Representative images (left) and quantification (right) of portal vein blood flow velocity in fibrotic livers treated as indicated (4-5 mice/group). (**J**-**K**) Analysis of alanine transaminase (ALT) (left) and aspartate transaminase (AST) (right) levels in CCl_4-induced fibrotic livers treated with free fucoidan, (J) free navitoclax, DexNav, or FiNav; (K) free dBET6, DexdBET6, or FidBET6 (4-5 mice/group). Each dot represents one mouse. Data are presented as mean ± s.e.m. One-way ANOVA and Sidak's multiple comparisons test used for all panels in this figure.

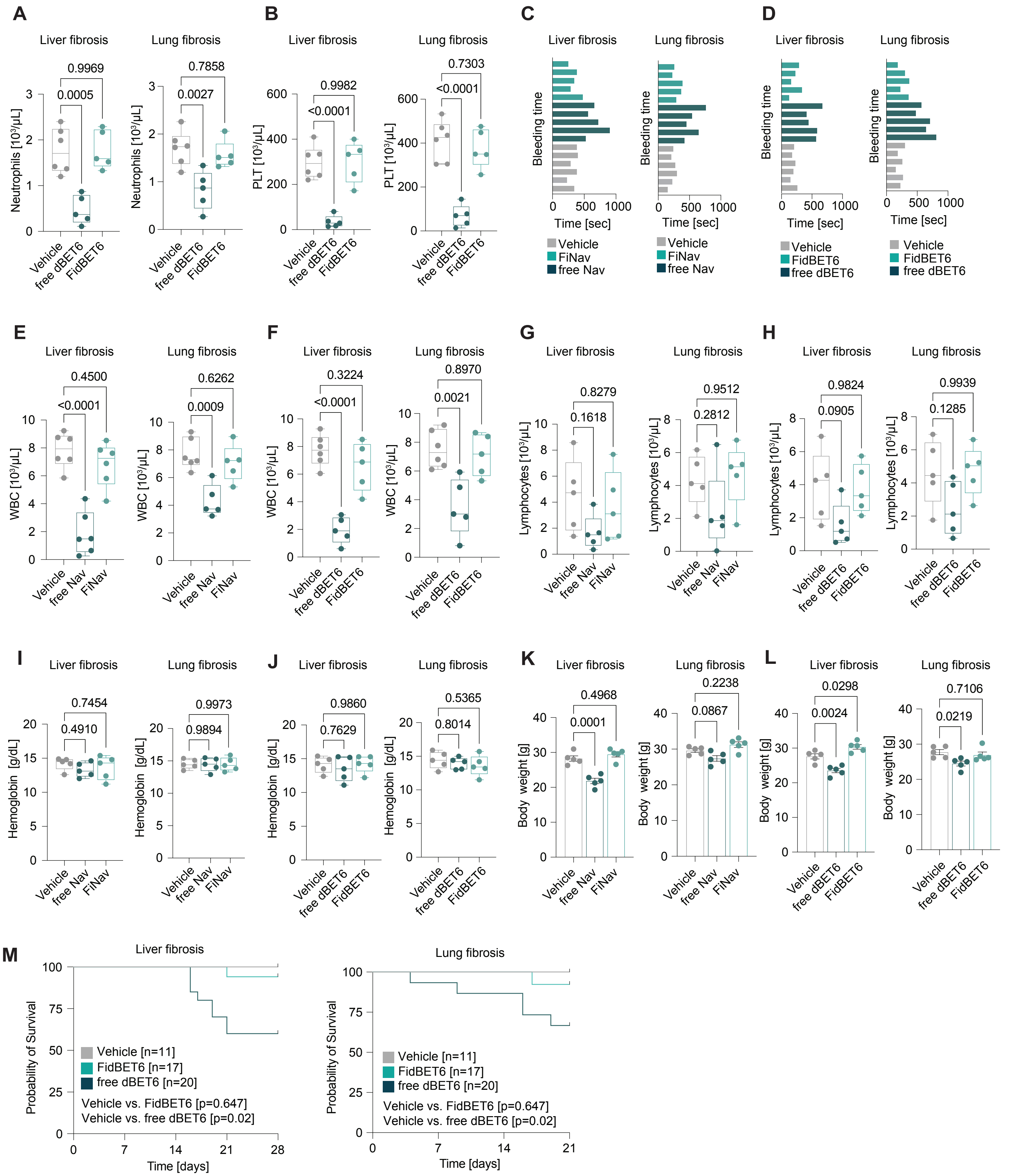
A
Liver fibrosis
Lung fibrosis
Neutrophils [10³/µL]
0.9969
0.0005
0.7858
0.0027
Vehicle
free dBET6
FidBET6
B
PLT [10³/µL]
0.9982
<0.0001
0.7303
C
Bleeding time
Time [sec]
Vehicle
FiNav
free Nav
D
FidBET6
free dBET6
E
WBC [10³/µL]
0.4500
0.6262
0.0009
F
0.3224
0.8970
0.0021
G
Lymphocytes [10³/µL]
0.8279
0.1618
0.9512
0.2812
H
0.9824
0.0905
0.9939
0.1285
I
Hemoglobin [g/dL]
0.7454
0.4910
0.9973
0.9894
J
0.9860
0.7629
0.5365
0.8014
K
Body weight [g]
0.4968
0.0001
0.2238
0.0867
L
0.0298
0.0024
0.7106
0.0219
M
Probability of Survival
Time [days]
Vehicle [n=11]
FidBET6 [n=17]
free dBET6 [n=20]
Vehicle vs. FidBET6 [p=0.647]
Vehicle vs. free dBET6 [p=0.02]

Figure S3: SMNPs eliminate drug-induced toxicities in mice with liver and lung fibrosis. (Related to Figure 2)

(**A**) Neutrophil counts in the blood of mice with fibrotic livers (left) and lungs (right) treated with free dBET6 or FidBET6 nanoparticles. (**B**) Platelet counts in the blood of mice with fibrotic livers (left) and (lungs) treated with free dBET6 or FidBET6. (**C-D**) Bleeding time upon clotting after tail vein injection of (C) free navitoclax or FiNav nanoparticles; (D) free dBET6 or FidBET6 nanoparticles in fibrotic livers (left) and lungs (right). (**E-F**) White blood cell (WBC) counts in serum of mice with fibrotic livers (left) and (lungs) treated with (E) free navitoclax or FiNav; (F) free dBET6 or FidBET6. (**G-H**) Lymphocyte counts in serum of mice with fibrotic livers (left) and (lungs) treated with (G) free navitoclax or FiNav; (H) free dBET6 or FidBET6. (**I-J**) Hemoglobin counts in serum of mice with fibrotic livers (left) and (lungs) treated with (I) free navitoclax or FiNav; (J) free dBET6 or FidBET6. (**K-L**) Body weight of mice with fibrotic livers (left) and lungs (right) treated with (K) free navitoclax or FiNav; (L) free dBET6 or FidBET6. (**M**) Kaplan-Meier analysis comparing overall survival of mice with fibrotic livers (left) or fibrotic lungs (right) treated with free dBET6 or FidBET6. All data were collected after 4 weeks of treatment in fibrotic livers and 3 weeks of treatment in fibrotic lungs (5-6 mice/group). Each dot or bar represents one mouse. Data are presented as mean ± s.e.m. One-way ANOVA with Sidak's multiple comparisons test used for (A-B, E-L), and log-rank Mantel-Cox test (M).

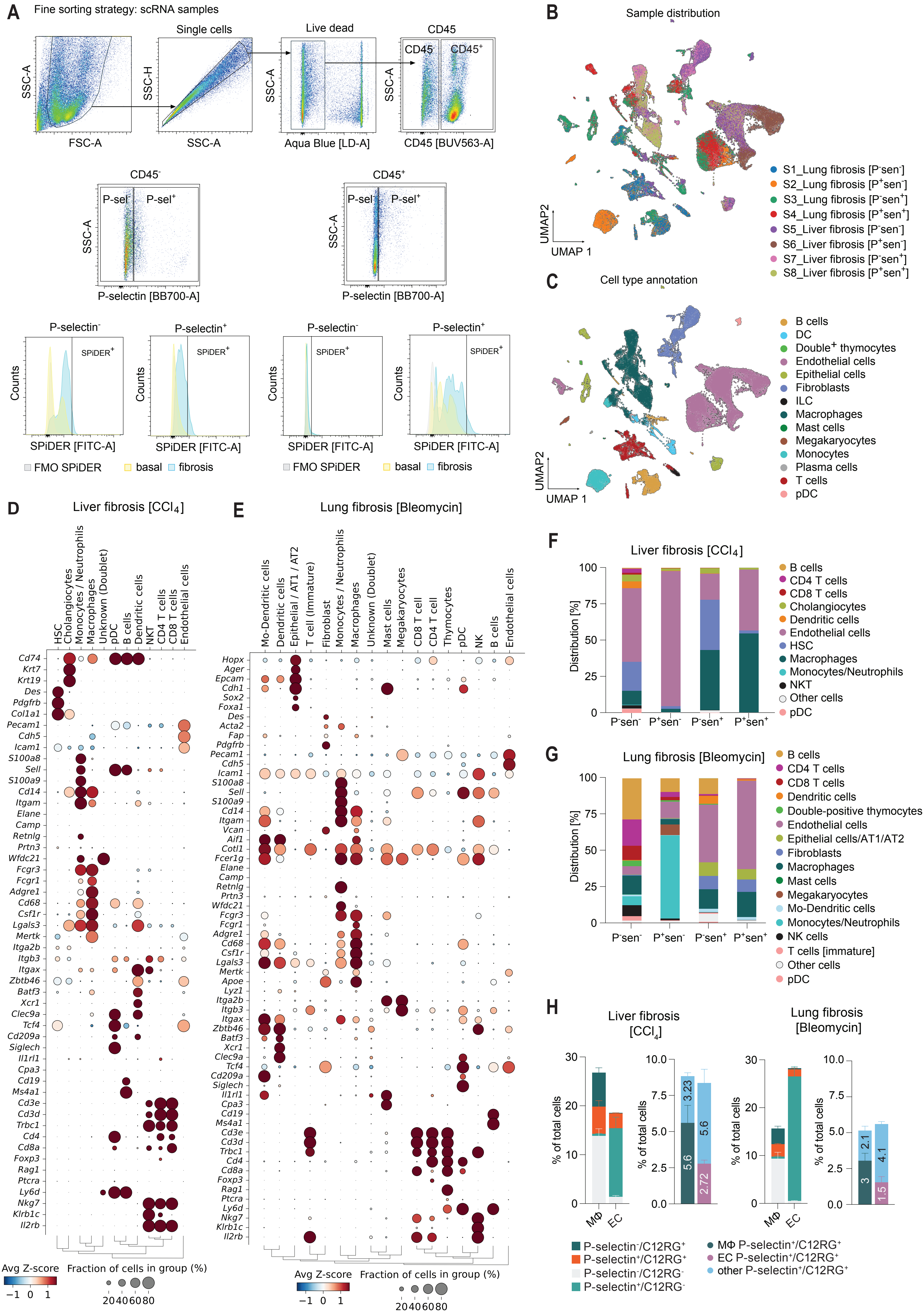

A
Fine sorting strategy: scRNA samples
SSC-A
FSC-A
Single cells
SSC-H
SSC-A
Live dead
SSC-A
Aqua Blue [LD-A]
CD45
CD45-
CD45+
SSC-A
CD45 [BUV563-A]
CD45-
CD45+
P-sel-
P-sel+
SSC-A
P-selectin [BB700-A]
P-selectin-
P-selectin+
SPiDER+
Counts
SPiDER [FITC-A]
FMO SPiDER
basal
fibrosis
B
Sample distribution
S1_Lung fibrosis [P-sen-]
S2_Lung fibrosis [P+sen-]
S3_Lung fibrosis [P-sen+]
S4_Lung fibrosis [P+sen+]
S5_Liver fibrosis [P-sen-]
S6_Liver fibrosis [P+sen-]
S7_Liver fibrosis [P+sen+]
S8_Liver fibrosis [P+sen+]
UMAP2
UMAP 1
C
Cell type annotation
B cells
DC
Double+ thymocytes
Endothelial cells
Epithelial cells
Fibroblasts
ILC
Macrophages
Mast cells
Megakaryocytes
Monocytes
Plasma cells
T cells
pDC
D
Liver fibrosis [CCl4]
HSC
Cholangiocytes
Monocytes / Neutrophils
Macrophages
Unknown (Doublet)
pDC
B cells
Dendritic cells
NKT
CD4 T cells
CD8 T cells
Endothelial cells
Cd74
Krt7
Krt19
Des
Pdgfrb
Col1a1
Pecam1
Cdh5
Icam1
S100a8
Sell
S100a9
Cd14
Itgam
Elane
Camp
Retnlg
Prtn3
Wfdc21
Fcgr3
Fcgr1
Adgre1
Cd68
Csf1r
Lgals3
Mertk
Itga2b
Itgb3
Itgax
Zbtb46
Batf3
Xcr1
Clec9a
Tcf4
Cd209a
Siglech
Il1rl1
Cpa3
Cd19
Ms4a1
Cd3e
Cd3d
Trbc1
Cd4
Cd8a
Foxp3
Rag1
Ptcra
Ly6d
Nkg7
Klrb1c
Il2rb
Avg Z-score
Fraction of cells in group (%)
−1 0 1
20 40 60 80
E
Lung fibrosis [Bleomycin]
Mo-Dendritic cells
Dendritic cells
Epithelial / AT1 / AT2
T cell (Immature)
Fibroblast
Monocytes / Neutrophils
Macrophages
Unknown (Doublet)
Mast cells
Megakaryocytes
CD8 T cell
CD4 T cell
Thymocytes
pDC
NK
B cells
Endothelial cells
Hopx
Ager
Epcam
Cdh1
Sox2
Foxa1
Des
Acta2
Fap
Pdgfrb
Pecam1
Cdh5
Icam1
S100a8
Sell
S100a9
Cd14
Itgam
Vcan
Aif1
Cotl1
Fcer1g
Elane
Camp
Retnlg
Prtn3
Wfdc21
Fcgr3
Fcgr1
Adgre1
Cd68
Csf1r
Lgals3
Mertk
Apoe
Lyz1
Itga2b
Itgb3
Itgax
Zbtb46
Batf3
Xcr1
Clec9a
Tcf4
Cd209a
Siglech
Il1rl1
Cpa3
Cd19
Ms4a1
Cd3e
Cd3d
Trbc1
Cd4
Cd8a
Foxp3
Rag1
Ptcra
Ly6d
Nkg7
Klrb1c
Il2rb
Avg Z-score
Fraction of cells in group (%)
−1 0 1
20 40 60 80
F
Liver fibrosis [CCl4]
Distribution [%]
100
75
50
25
0
P-sen-
P+sen-
P-sen+
P+sen+
B cells
CD4 T cells
CD8 T cells
Cholangiocytes
Dendritic cells
Endothelial cells
HSC
Macrophages
Monocytes/Neutrophils
NKT
Other cells
pDC
G
Lung fibrosis [Bleomycin]
Distribution [%]
100
75
50
25
0
P-sen-
P+sen-
P-sen+
P+sen+
B cells
CD4 T cells
CD8 T cells
Dendritic cells
Double-positive thymocytes
Endothelial cells
Epithelial cells/AT1/AT2
Fibroblasts
Macrophages
Mast cells
Megakaryocytes
Mo-Dendritic cells
Monocytes/Neutrophils
NK cells
T cells [immature]
Other cells
pDC
H
Liver fibrosis [CCl4]
Lung fibrosis [Bleomycin]
% of total cells
30
20
10
0
MΦ
EC
10.0
7.5
5.0
2.5
0.0
5.6
3.23
2.72
5.6
3
2.1
1.5
4.1
P-selectin-/C12RG+
P-selectin+/C12RG+
P-selectin-/C12RG-
P-selectin+/C12RG-
MΦ P-selectin+/C12RG+
EC P-selectin+/C12RG+
other P-selectin+/C12RG+

Figure S4: Characterization of P-selectin positive states in fibrotic liver and lungs. (Related to Figure 3)

(**A**) Gating strategy for the isolation of live $CD45^+$ and $CD45^-$ populations based on P-selectin surface expression and b-galactosidase activity (SPiDER) in healthy and fibrotic tissues. (**B**) Visualization of unsupervised clustering in a UMAP of cells isolated from murine fibrotic liver and lung, sorted as in (A), then subjected to scRNA-seq. (**C**) Cell type annotations of the UMAP in (B). (**D-E**) Dot plots showing expression levels of representative marker genes within each cell population from fibrotic liver (D) and lung (E). (**F-G**) Proportions of the indicated cell types across sorted samples in fibrotic livers (F) and lungs (G). (**H**) (Left) Proportion of P-selectin$^-$/C12RG$^+$, P-selectin$^+$/C12RG$^+$, P-selectin$^+$/C12RG$^-$, and P-selectin$^-$/C12RG$^-$ macrophages (MΦ) and endothelial cells (EC) from sorted samples from fibrotic livers and lungs. (Right) Proportion of P-selectin$^+$/C12RG$^+$ MΦ, P-selectin$^+$/C12RG$^+$ EC, and other P-selectin$^+$/C12RG$^+$ cells in fibrotic livers and lungs (related to Fig. 3E-F).

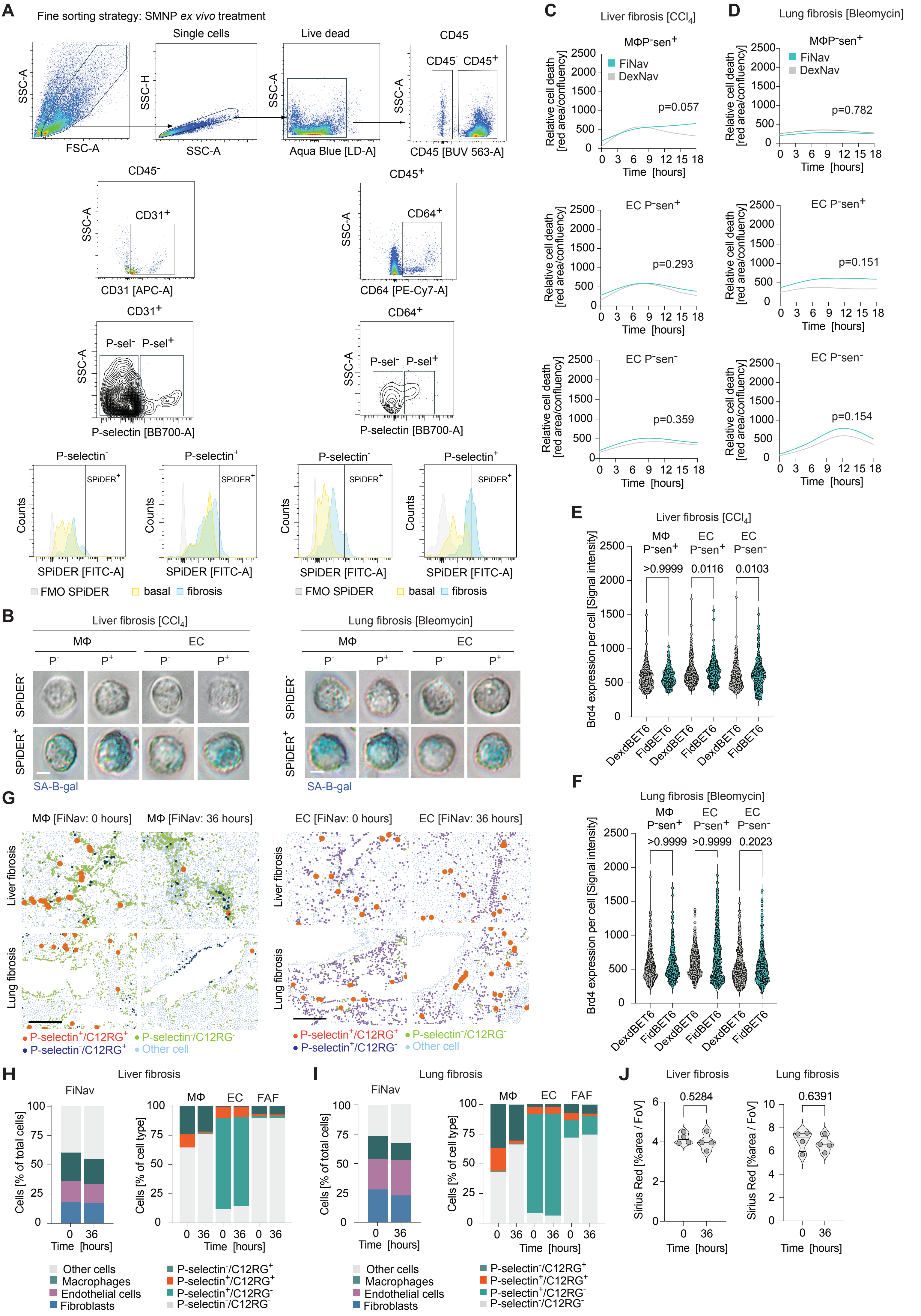

A
Fine sorting strategy: SMNP ex vivo treatment
SSC-A
FSC-A
Single cells
SSC-H
SSC-A
Live dead
SSC-A
Aqua Blue [LD-A]
CD45
CD45⁻ CD45⁺
SSC-A
CD45 [BUV 563-A]
CD45⁻
SSC-A
CD31⁺
CD31 [APC-A]
CD45⁺
SSC-A
CD64⁺
CD64 [PE-Cy7-A]
CD31⁺
SSC-A
P-sel⁻ P-sel⁺
P-selectin [BB700-A]
CD64⁺
SSC-A
P-sel⁻ P-sel⁺
P-selectin [BB700-A]
P-selectin⁻
P-selectin⁺
P-selectin⁻
P-selectin⁺
Counts
SPiDER⁺
SPiDER [FITC-A]
FMO SPiDER
basal
fibrosis
C
Liver fibrosis [CCl4]
MΦP⁻sen⁺
FiNav
DexNav
p=0.057
EC P⁻sen⁺
p=0.293
EC P⁻sen⁻
p=0.359
Relative cell death [red area/confluency]
Time [hours]
0 3 6 9 12 15 18
D
Lung fibrosis [Bleomycin]
MΦP⁻sen⁺
FiNav
DexNav
p=0.782
EC P⁻sen⁺
p=0.151
EC P⁻sen⁻
p=0.154
Relative cell death [red area/confluency]
Time [hours]
B
Liver fibrosis [CCl4]
MΦ
EC
P⁻
P⁺
SPiDER⁻
SPiDER⁺
SA-B-gal
Lung fibrosis [Bleomycin]
E
Liver fibrosis [CCl4]
MΦ P⁻sen⁺
EC P⁻sen⁺
EC P⁻sen⁻
>0.9999
0.0116
0.0103
Brd4 expression per cell [Signal intensity]
DexdBET6
FidBET6
F
Lung fibrosis [Bleomycin]
MΦ P⁻sen⁺
EC P⁻sen⁺
EC P⁻sen⁻
>0.9999
>0.9999
0.2023
Brd4 expression per cell [Signal intensity]
DexdBET6
FidBET6
G
MΦ [FiNav: 0 hours]
MΦ [FiNav: 36 hours]
EC [FiNav: 0 hours]
EC [FiNav: 36 hours]
Liver fibrosis
Lung fibrosis
P-selectin⁺/C12RG⁺
P-selectin⁻/C12RG⁻
P-selectin⁻/C12RG⁺
Other cell
P-selectin⁺/C12RG⁺
P-selectin⁻/C12RG⁻
P-selectin⁺/C12RG⁻
Other cell
H
Liver fibrosis
FiNav
Cells [% of total cells]
Time [hours]
0 36
MΦ EC FAF
Cells [% of cell type]
Other cells
Macrophages
Endothelial cells
Fibroblasts
P-selectin⁻/C12RG⁺
P-selectin⁺/C12RG⁺
P-selectin⁺/C12RG⁻
P-selectin⁻/C12RG⁻
I
Lung fibrosis
FiNav
Cells [% of total cells]
Time [hours]
MΦ EC FAF
Cells [% of cell type]
Other cells
Macrophages
Endothelial cells
Fibroblasts
P-selectin⁻/C12RG⁺
P-selectin⁺/C12RG⁺
P-selectin⁺/C12RG⁻
P-selectin⁻/C12RG⁻
J
Liver fibrosis
0.5284
Sirius Red [%area / FoV]
Time [hours]
Lung fibrosis
0.6391
Sirius Red [%area / FoV]
Time [hours]

Figure S5: Functional assessment of SMNP target populations in fibrotic liver and lungs. (Related to Figure 3)

(**A**) Gating strategy for the isolation of $CD31^+$ endothelial cells (EC) (left) and $CD64^+$ macrophages (MΦ) (right) based on their P-selectin expression and SA-b-galactosidase activity (SPiDER) in healthy and fibrotic murine lungs. (**B**) Representative images of senescence-associated β-galactosidase activity in the different sorted population of EC and MΦ from (A). Scale bars, 5 µm. (**C-D**) Ex-vivo killing assays of sorted P-selectin$^-$ senescent$^+$ (P-sen$^+$) MΦ, P$^-$sen$^+$ EC, or P-selectin$^-$ senescent$^-$ (P$^-$sen$^-$) EC from murine fibrotic livers (C) and lungs (D) treated with DexNav or FiNav nanoparticles. Representative data from three independent experiments. (**E-F**) Quantification of Brd4 protein after DexdBET6 or FidBET6 treatment of P$^-$sen$^+$MΦ, P$^-$sen$^+$ EC, and P$^-$sen$^-$ ECs from murine fibrotic livers (E) and lungs (F). Data are from four independent biological replicates and presented as mean ± s.e.m. (**G**) Representative images of mIF analysis of P-selectin$^+$/C12RG$^+$, P-selectin$^-$/C12RG$^+$, and P-selectin$^-$/C12RG$^-$ MΦ (left) and ECs (right) from fibrotic livers (E) and lungs (F) before (0 hour) and after (36 hours) FiNav injection. Scale bars, 250 µm. (**H-I**) Proportions of various cell types from fibrotic livers (H) and lungs (I) before (0 hour) and after (36 hours) FiNav injection. (Left) Macrophages, endothelial cells, and fibroblasts; (right) P-selectin$^+$/C12RG$^+$, P-selectin$^-$/C12RG$^+$, P-selectin$^+$/C12RG$^-$, and P-selectin$^-$/C12RG$^-$ MΦ, ECs, and fibroblasts (FAF) (4 mice/group). (**J**) Quantification of collagen deposition (Sirius Red) in fibrotic livers (left) and lungs (right) before (0 hour) and after (36 hours) FiNav injection (4 mice/group). Each dot represents one cell. Statistical tests were Mann-Whitney test or Welch's t-test at endpoint (18h) for (C,D), Mann-Whitney test for (E,F), and unpaired t-test were used for (J).

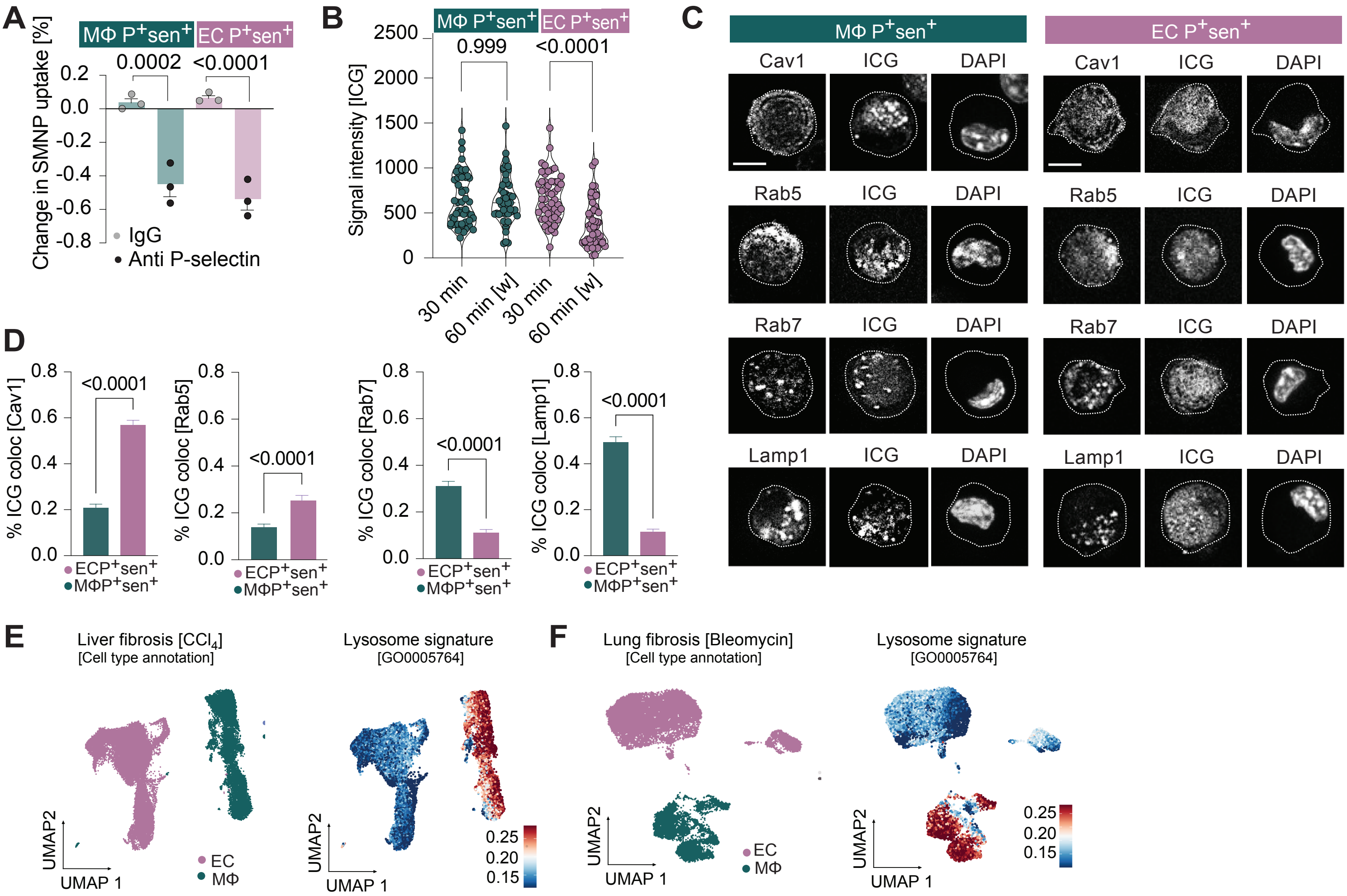

A
MΦ P+sen+
EC P+sen+
0.0002
<0.0001
Change in SMNP uptake [%]
IgG
Anti P-selectin
B
MΦ P+sen+
EC P+sen+
0.999
<0.0001
Signal intensity [ICG]
30 min
60 min [w]
30 min
60 min [w]
C
MΦ P+sen+
EC P+sen+
Cav1
ICG
DAPI
Rab5
Rab7
Lamp1
D
% ICG coloc [Cav1]
% ICG coloc [Rab5]
% ICG coloc [Rab7]
% ICG coloc [Lamp1]
<0.0001
ECP+sen+
MΦP+sen+
E
Liver fibrosis [CCl4]
[Cell type annotation]
Lysosome signature
[GO0005764]
UMAP2
UMAP 1
EC
MΦ
F
Lung fibrosis [Bleomycin]
[Cell type annotation]

Figure S6: Characterization of uptake and intracellular trafficking of SMNPs. (Related to Figure 3)

(**A**) SMNP (FidBET6) uptake in sorted P^+sen^+ macrophages (MΦ) and endothelial cells (EC) from murine fibrotic lungs following 2-hour treatment with FidBET6 nanoparticles in combination with P-selectin blocking antibody or IgG control. Change in NP uptake is relative to IgG controls. (**B**) Quantification of nanoparticle signal (ICG) in sorted P^+sen^+ EC and MΦ from murine fibrotic lungs after either a 30-minute exposure to FidBET6 nanoparticles or 30 minutes of exposure followed by a 30-minute washout (60 min [w]). (**C**) Representative IF images of nanoparticle signal (ICG) with vesicular compartment markers: Cav1 (transcytosis-associated marker), Rab5 (early endosomes), Rab7 (late endosomes), and Lamp1 (lysosomes) in sorted P^+sen^+ EC and MΦ from murine fibrotic lungs following treatment with FidBET6 nanoparticles. (**D**) Quantification of ICG^+ SMNP colocalization with Cav1 (far left), Rab5 (left), Rab7 (right) or Lamp1 (far right) from images in C. (**E-F**) Lysosome signature in ECs and MΦ sorted from murine fibrotic liver (E) and lung (F). Left, cell type annotations in a UMAP of ECs and MΦ; right, enrichment of signatures for lysosomes. Each dot represents the mean signal across replicates (A), or one cell (B). (A and D) Data are presented as mean ± s.e.m. Mann Whitney test was used for (A-B, D).

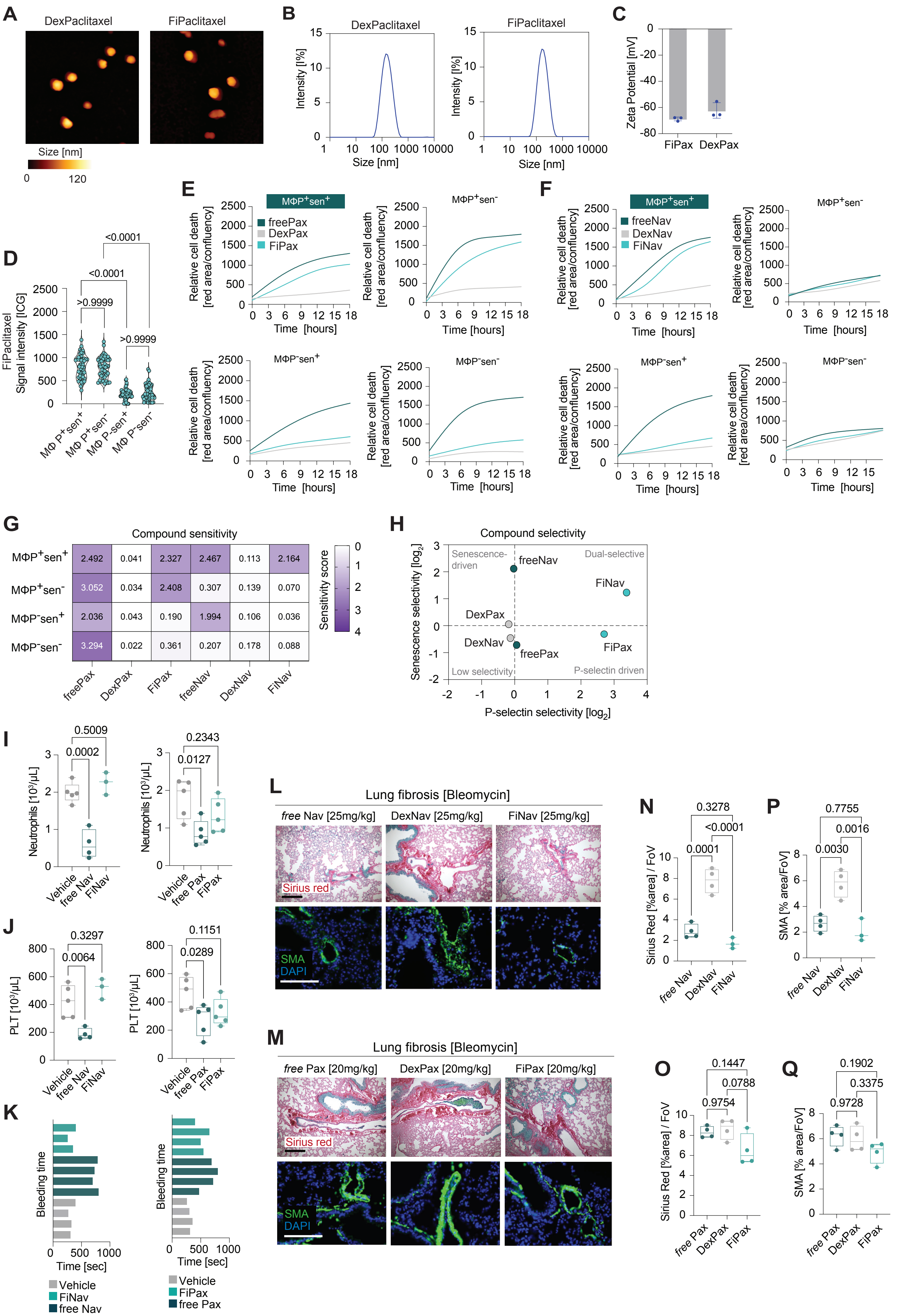
A
DexPaclitaxel
FiPaclitaxel
Size [nm]
0
120
B
DexPaclitaxel
FiPaclitaxel
Intensity [%]
Size [nm]
C
Zeta Potential [mV]
FiPax
DexPax
D
FiPaclitaxel Signal intensity [ICG]
<0.0001
<0.0001
>0.9999
>0.9999
MΦ P+sen+
MΦ P+sen-
MΦ P-sen+
MΦ P-sen-
E
MΦP+sen+
MΦP+sen-
MΦP-sen+
MΦP-sen-
freePax
DexPax
FiPax
Relative cell death [red area/confluency]
Time [hours]
F
MΦP+sen+
MΦP+sen-
MΦP-sen+
MΦP-sen-
freeNav
DexNav
FiNav
Relative cell death [red area/confluency]
Time [hours]
G
Compound sensitivity
MΦP+sen+ 2.492 0.041 2.327 2.467 0.113 2.164
MΦP+sen- 3.052 0.034 2.408 0.307 0.139 0.070
MΦP-sen+ 2.036 0.043 0.190 1.994 0.106 0.036
MΦP-sen- 3.294 0.022 0.361 0.207 0.178 0.088
freePax DexPax FiPax freeNav DexNav FiNav
Sensitivity score
H
Compound selectivity
Senescence-driven
Dual-selective
Low selectivity
P-selectin driven
freeNav
FiNav
DexPax
DexNav
freePax
FiPax
Senescence selectivity [log2]
P-selectin selectivity [log2]
I
Neutrophils [10³/µL]
0.5009
0.0002
Vehicle
free Nav
FiNav
0.2343
0.0127
Vehicle
free Pax
FiPax
J
PLT [10³/µL]
0.3297
0.0064
Vehicle
free Nav
FiNav
0.1151
0.0289
Vehicle
free Pax
FiPax
K
Bleeding time
Time [sec]
Vehicle
FiNav
free Nav
Vehicle
FiPax
free Pax
L
Lung fibrosis [Bleomycin]
free Nav [25mg/kg]
DexNav [25mg/kg]
FiNav [25mg/kg]
Sirius red
SMA
DAPI
M
Lung fibrosis [Bleomycin]
free Pax [20mg/kg]
DexPax [20mg/kg]
FiPax [20mg/kg]
Sirius red
SMA
DAPI
N
Sirius Red [%area] / FoV
0.3278
<0.0001
0.0001
free Nav
DexNav
FiNav
P
SMA [% area/FoV]
0.7755
0.0016
0.0030
free Nav
DexNav
FiNav
O
Sirius Red [%area] / FoV
0.1447
0.0788
0.9754
free Pax
DexPax
FiPax
Q
SMA [% area/FoV]
0.1902
0.3375
0.9728
free Pax
DexPax
FiPax

Figure S7: Characterization and activity of non-senotherapeutic nanoparticles. (Related to Figure 3)

(**A**) Representative atomic force microscopy images used to assess the size of paclitaxel-encapsulating dextran nanoparticles (DexPax) and fucoidan nanoparticles (FiPax). (**B**) Dynamic light scattering size distribution from representative individual nanoprecipitations of DexPax (left) and FiPax (right). (**C**) Surface zeta potential confirming dextran and fucoidan surface functionalization (n=3 nanoprecipitation samples, 1 experiment). (**D**) Quantification of nanoparticles (ICG) in sorted P^+sen^+, P^+sen^-, P^-sen^+, and P^-sen^- MΦ from murine fibrotic lungs following treatment with FiPax. (**E-F**) Ex-vivo killing of sorted P^+sen^+, P^+sen^-, P^-sen^+, and P^-sen^- MΦs from murine fibrotic lungs treated with (E) free Paclitaxel (Pax), DexPax, or FiPax and (F) free Nav, DexNav, or FiNav. (**G**) Sensitivity table of freePax, DexPax, FiPax, freeNav, DexNav, and FiNav drugs for the indicated macrophage populations in fibrotic lungs. (**H**) Selectivity of free Pax, DexPax, FiPax, free Nav, DexNav, and FiNav for senescence, P-selectin, or both in fibrotic lungs. (**I-J**) Neutrophil (I) and platelet (J) counts from blood of mice with fibrotic lungs treated with (left) free navitoclax or FiNav and (right) free Paclitaxel or FiPax (3-5 mice/group). (**K**) Bleeding time upon clotting after tail vein injection of (left) free navitoclax or FiNav, and (right) free Paclitaxel or FiPax in fibrotic lungs. (**L-M**) Representative images of collagen deposition (Sirius Red), and fibroblast activation (SMA), in fibrotic lungs treated with (L) free Nav, DexNav, or FiNav or (M) free Pax, DexPax, or FiPax. Scale bars, 150 μm. (**N-O**) Quantification of collagen deposition (Sirius Red) in fibrotic lungs treated with (N) free Nav, DexNav, or FiNav or (O) free Pax, DexPax, or FiPax (3-4 mice/group). (**P-Q**) Quantification of myofibroblast activation in fibrotic lungs treated with (P) free Nav, DexNav, or FiNav or (Q) free Pax, DexPax, or FiPax (3-4 mice/group). Each dot represents one independent nanoparticle batch generated (C), one cell (D), or one mouse (I-J,N-Q). Each bar represents one mouse. (C-D, I-J, N-Q) Data are presented as mean ± s.e.m. One-way ANOVA with Sidak's multiple comparisons test was used for (D, I-J, N-Q).

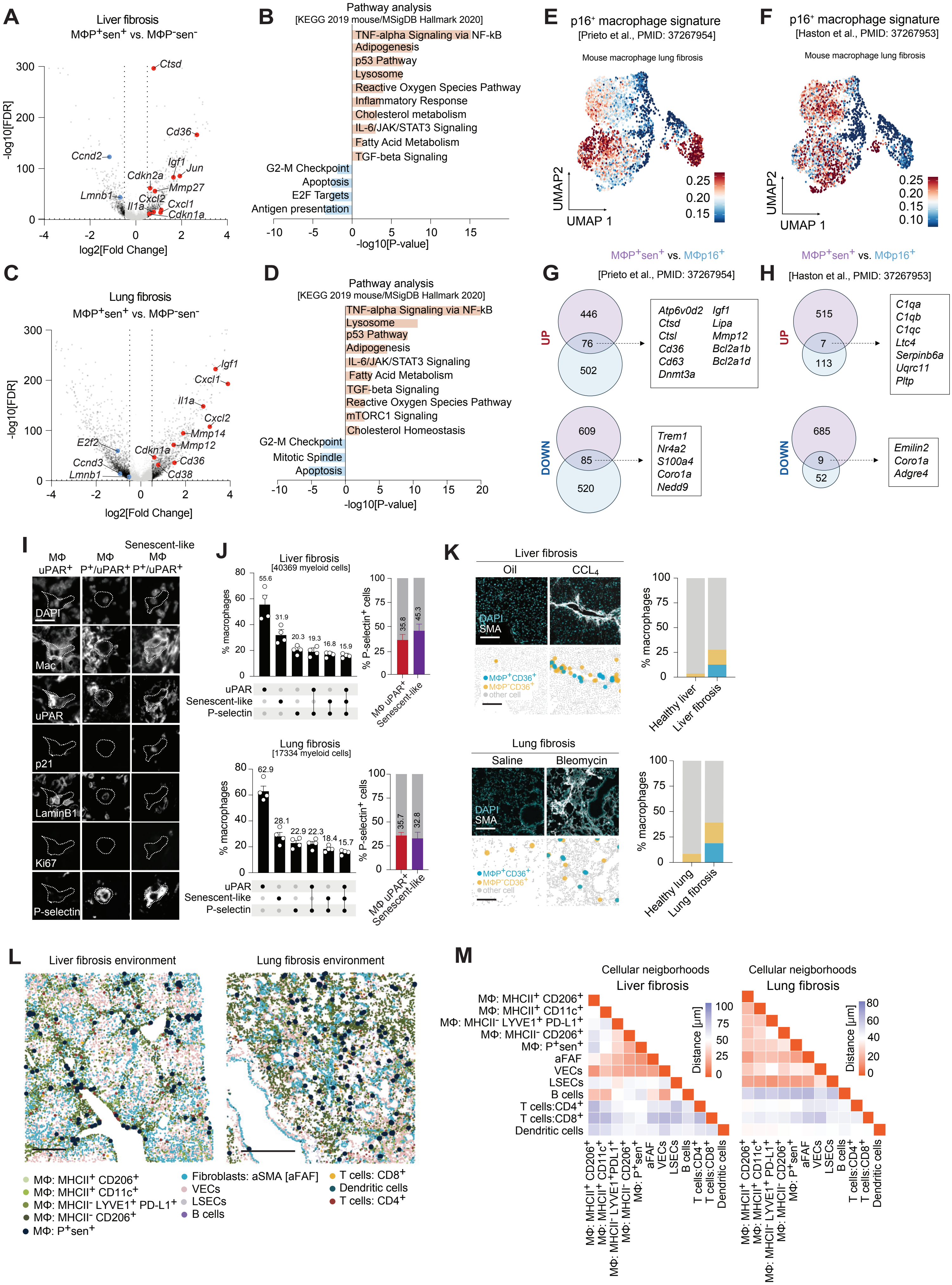

A
Liver fibrosis
MΦP+sen+ vs. MΦP-sen-
-log10[FDR]
log2[Fold Change]
Ctsd
Cd36
Ccnd2
Igf1
Jun
Cdkn2a
Mmp27
Lmnb1
Cxcl2
Il1a
Cxcl1
Cdkn1a
B
Pathway analysis
[KEGG 2019 mouse/MSigDB Hallmark 2020]
TNF-alpha Signaling via NF-kB
Adipogenesis
p53 Pathway
Lysosome
Reactive Oxygen Species Pathway
Inflammatory Response
Cholesterol metabolism
IL-6/JAK/STAT3 Signaling
Fatty Acid Metabolism
TGF-beta Signaling
G2-M Checkpoint
Apoptosis
E2F Targets
Antigen presentation
-log10[P-value]
C
Lung fibrosis
MΦP+sen+ vs. MΦP-sen-
Igf1
Cxcl1
Il1a
Cxcl2
Mmp14
Mmp12
E2f2
Cdkn1a
Cd36
Ccnd3
Lmnb1
Cd38
D
Pathway analysis
[KEGG 2019 mouse/MSigDB Hallmark 2020]
TNF-alpha Signaling via NF-kB
Lysosome
p53 Pathway
Adipogenesis
IL-6/JAK/STAT3 Signaling
Fatty Acid Metabolism
TGF-beta Signaling
Reactive Oxygen Species Pathway
mTORC1 Signaling
Cholesterol Homeostasis
G2-M Checkpoint
Mitotic Spindle
Apoptosis
E
p16+ macrophage signature
[Prieto et al., PMID: 37267954]
Mouse macrophage lung fibrosis
UMAP2
UMAP 1
F
p16+ macrophage signature
[Haston et al., PMID: 37267953]
Mouse macrophage lung fibrosis
G
MΦP+sen+ vs. MΦp16+
[Prieto et al., PMID: 37267954]
UP
446
76
502
Atp6v0d2 Igf1
Ctsd Lipa
Ctsl Mmp12
Cd36 Bcl2a1b
Cd63 Bcl2a1d
Dnmt3a
DOWN
609
85
520
Trem1
Nr4a2
S100a4
Coro1a
Nedd9
H
MΦP+sen+ vs. MΦp16+
[Haston et al., PMID: 37267953]
515
7
113
C1qa
C1qb
C1qc
Ltc4
Serpinb6a
Uqrc11
Pltp
685
9
52
Emilin2
Coro1a
Adgre4
I
MΦ uPAR+
MΦ P+/uPAR+
Senescent-like MΦ P+/uPAR+
DAPI
Mac
uPAR
p21
LaminB1
Ki67
P-selectin
J
Liver fibrosis
[40369 myeloid cells]
% macrophages
55.6
31.9
20.3
19.3
16.8
15.9
uPAR
Senescent-like
P-selectin
% P-selectin+ cells
35.8
45.3
MΦ uPAR+
Senescent-like
Lung fibrosis
[17334 myeloid cells]
62.9
28.1
22.9
22.3
18.4
15.7
35.7
32.8
K
Liver fibrosis
Oil
CCL4
DAPI
SMA
MΦP+CD36+
MΦP-CD36+
other cell
Healthy liver
Liver fibrosis
Lung fibrosis
Saline
Bleomycin
Healthy lung
Lung fibrosis
L
Liver fibrosis environment
Lung fibrosis environment
MΦ: MHCII+ CD206+
MΦ: MHCII+ CD11c+
MΦ: MHCII- LYVE1+ PD-L1+
MΦ: MHCII- CD206+
MΦ: P+sen+
Fibroblasts: aSMA [aFAF]
VECs
LSECs
B cells
T cells: CD8+
Dendritic cells
T cells: CD4+
M
Cellular neigborhoods
Liver fibrosis
Lung fibrosis
Distance [µm]
aFAF
VECs
LSECs
B cells
T cells:CD4+
T cells:CD8+
Dendritic cells

Figure S8: Transcriptional and spatial characterization of P+sen+ macrophages. (Related to Figure 4)

(**A**) Volcano plot showing genes differentially expressed in P^+sen^+ macrophages compared to P^-sen^- macrophages in liver fibrosis. Representative senescence-associated genes are highlighted. (**B**) Upregulated and downregulated pathways of P^+sen^+ macrophages vs P^-sen^- macrophages in murine fibrotic livers. (**C**) Volcano plot showing genes differentially expressed in P^+sen^+ macrophages compared to P^-sen^- macrophages in lung fibrosis. Representative senescence-associated genes are highlighted. (**D**) Upregulated and downregulated pathways of P^+sen^+ macrophages vs P^-sen^- macrophages in murine fibrotic lungs. (**E-F**) Gene signature analysis of P^+sen^+ macrophages compared to P^-sen^- macrophages. The UMAP plots show enrichment of signatures for $p16^+$ macrophages in murine lung fibrosis. (**G-H**) Venn diagrams representing the common upregulated (UP) or downregulated (DOWN) genes (derived from scRNA data) between P^+sen^+ macrophages compared to $p16^+$ macrophages in murine lung fibrosis. (**I**) mIF analysis of uPAR, p21, LaminB1, Ki67, and P-selectin expression in macrophages from fibrotic murine livers. Senescent-like phenotype is defined by p21-positivity and absence of Ki67 and LaminB1 expression. (**J**) (Left) Quantification of uPAR, senescent-like, and/or P-selectin expression in macrophages and (right) quantification of uPAR and senescent-like expression in P-selectin$^+$ myeloid cells of murine liver (top) and lung (bottom) fibrosis. (**K**) (Left) Representative images of mIF analysis mapping $CD36^+$ and P-selectin$^+$/$CD36^+$ macrophages and (right) quantification of P^+CD36^+ and P^-CD36^+ macrophages among total macrophages of healthy and fibrotic murine liver (top) and lung (bottom). Scale bars, 250 µm. (**L**) Representative images of mIF analysis mapping different immune and stromal populations in murine fibrotic livers (left) and lungs (right). Scale bars, 250 µm. VEC: vascular endothelial cells; LSEC: Liver sinusoidal endothelial cells. (**M**) Cellular neighborhood analysis measuring the distance between the indicated immune and stromal populations in murine fibrotic livers (left) and lungs (right). Each dot represents one mouse (J).

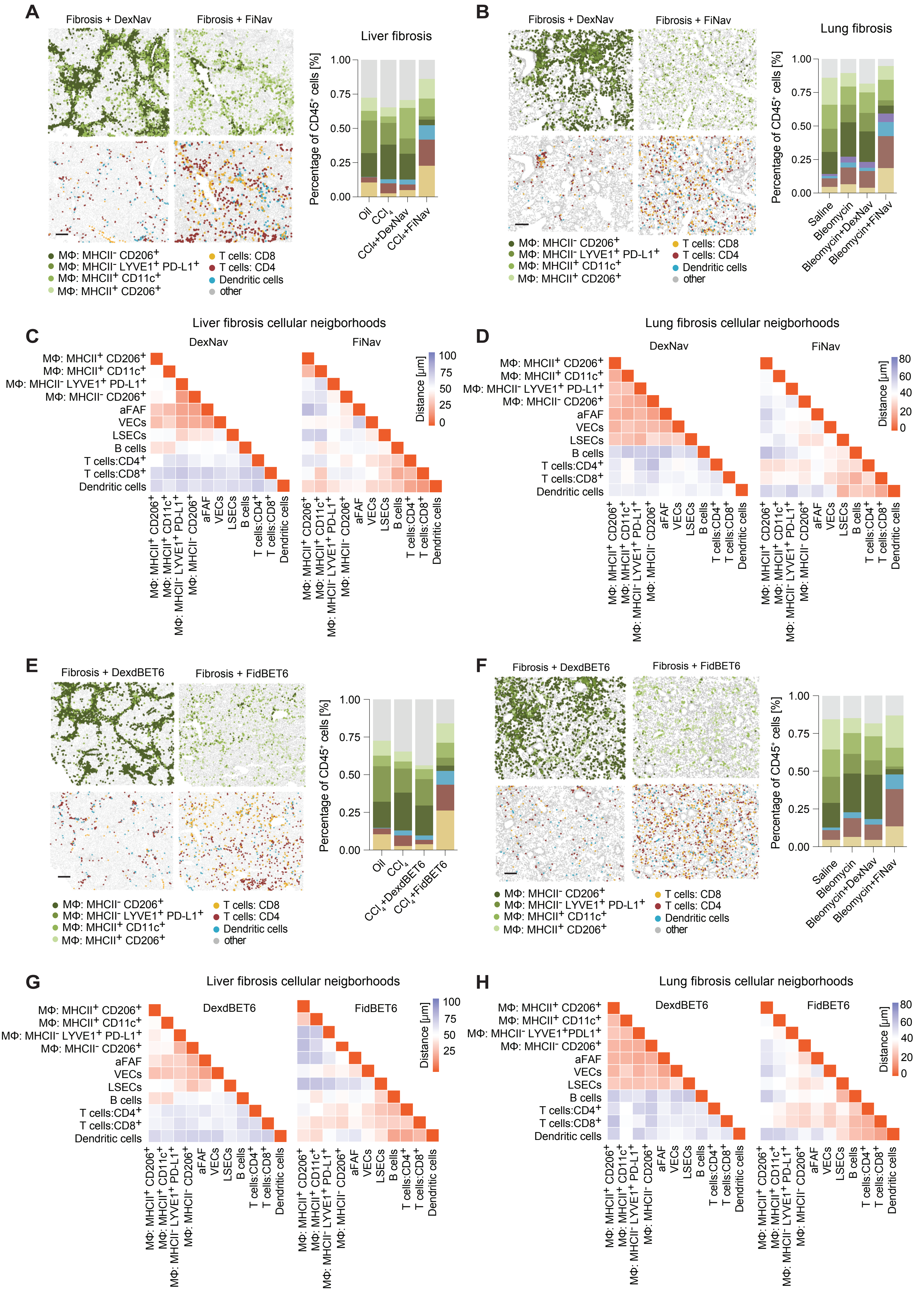

A
Fibrosis + DexNav
Fibrosis + FiNav
Liver fibrosis
Percentage of CD45+ cells [%]
1.00
0.75
0.50
0.25
0.00
Oil
CCl4
CCl4+DexNav
CCl4+FiNav
MΦ: MHCII- CD206+
MΦ: MHCII- LYVE1+ PD-L1+
MΦ: MHCII+ CD11c+
MΦ: MHCII+ CD206+
T cells: CD8
T cells: CD4
Dendritic cells
other
B
Fibrosis + DexNav
Fibrosis + FiNav
Lung fibrosis
Percentage of CD45+ cells [%]
Saline
Bleomycin
Bleomycin+DexNav
Bleomycin+FiNav
C
Liver fibrosis cellular neigborhoods
DexNav
FiNav
Distance [µm]
100
75
50
25
0
MΦ: MHCII+ CD206+
MΦ: MHCII+ CD11c+
MΦ: MHCII- LYVE1+ PD-L1+
MΦ: MHCII- CD206+
aFAF
VECs
LSECs
B cells
T cells:CD4+
T cells:CD8+
Dendritic cells
D
Lung fibrosis cellular neigborhoods
DexNav
FiNav
Distance [µm]
80
60
40
20
0
E
Fibrosis + DexdBET6
Fibrosis + FidBET6
CCl4+DexdBET6
CCl4+FidBET6
F
Fibrosis + DexdBET6
Fibrosis + FidBET6
G
Liver fibrosis cellular neigborhoods
DexdBET6
FidBET6
H
Lung fibrosis cellular neigborhoods
DexdBET6
FidBET6
MΦ: MHCII- LYVE1+PDL1+

Figure S9: Spatial characterization of the immune microenvironment remodeling in fibrotic tissues upon SMNP treatment. (Related to Figure 4)
(**A-B**) (Left) Representative images of mIF analysis mapping immune and stromal populations in murine fibrotic livers (A) and lungs (B) treated for four weeks with DexNav or FiNav nanoparticles. Scale bars, 250 µm. (Right) Quantification of the indicated immune and stomal populations. (**C-D**) Cellular neighborhood analysis measuring the distance between the indicated immune and stromal populations in murine fibrotic livers (C) and lungs (D) treated for four weeks with DexNav or FiNav. (**E-F**) (Left) Representative images of mIF analysis mapping immune and stromal populations in murine fibrotic livers (E) and lungs (F) treated for four weeks with DexdBET6 or FidBET6. Scale bars, 100 µm. (Right) Quantification of the indicated immune and stomal populations. (**G-H**) Cellular neighborhood analysis measuring the distance between the indicated immune and stromal populations in murine fibrotic livers (C) and lungs (D) treated for four weeks with DexdBET6 or FidBET6.

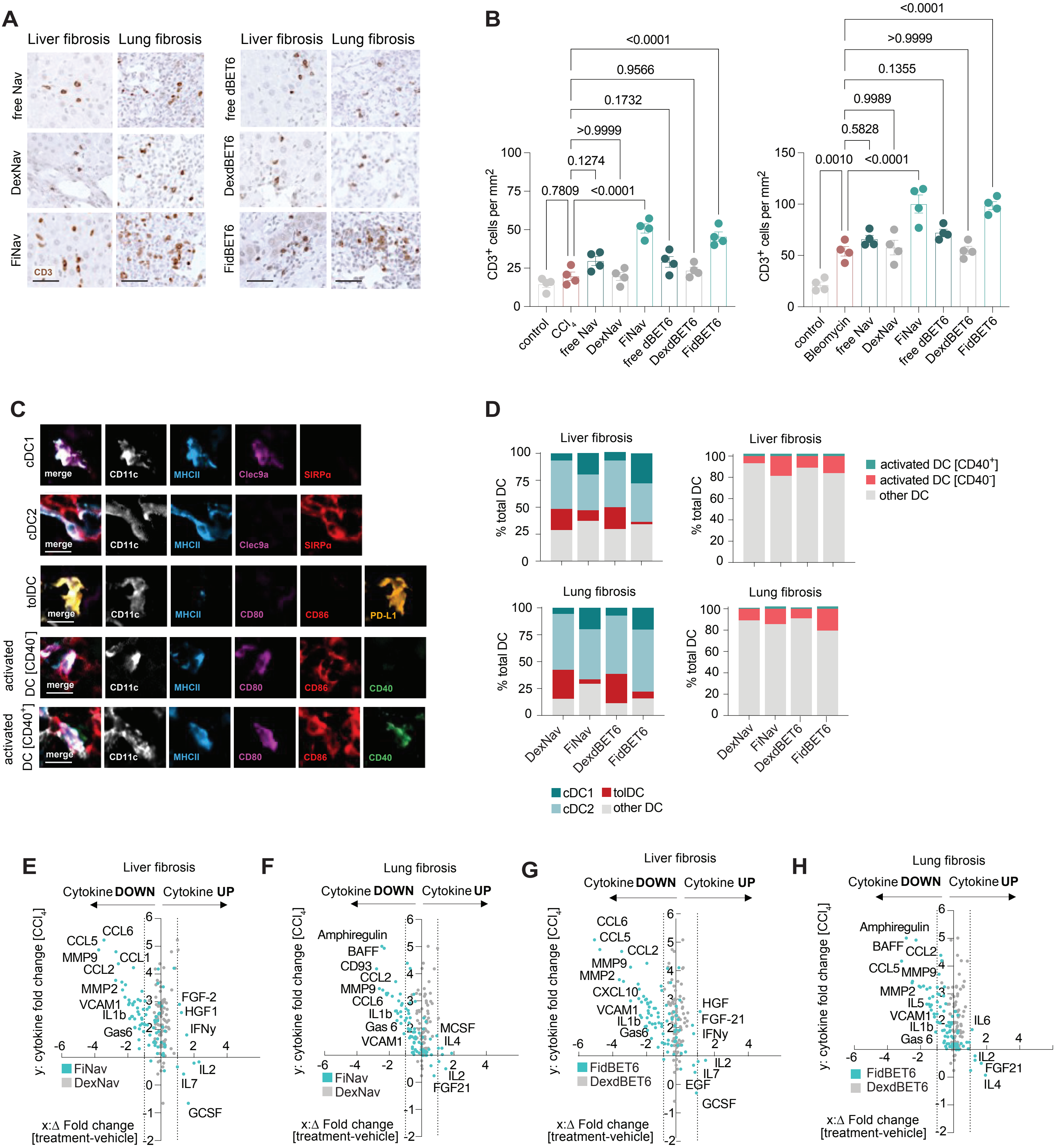

A
Liver fibrosis
Lung fibrosis
free Nav
DexNav
FiNav
CD3
free dBET6
DexdBET6
FidBET6
B
CD3+ cells per mm2
control
CCl4
free Nav
DexNav
FiNav
free dBET6
DexdBET6
FidBET6
<0.0001
0.9566
0.1732
>0.9999
0.1274
0.7809
<0.0001
Bleomycin
<0.0001
>0.9999
0.1355
0.9989
0.5828
0.0010
<0.0001
C
cDC1
cDC2
tolDC
activated DC [CD40-]
activated DC [CD40+]
merge
CD11c
MHCII
Clec9a
SIRPα
CD80
CD86
PD-L1
CD40
D
Liver fibrosis
Lung fibrosis
% total DC
DexNav
FiNav
DexdBET6
FidBET6
activated DC [CD40+]
activated DC [CD40-]
other DC
cDC1
toIDC
cDC2
other DC
E
Liver fibrosis
Cytokine DOWN
Cytokine UP
y: cytokine fold change [CCl4]
x:Δ Fold change [treatment-vehicle]
CCL6
CCL5
MMP9
CCL1
CCL2
MMP2
VCAM1
IL1b
Gas6
FGF-2
HGF1
IFNy
IL2
IL7
GCSF
FiNav
DexNav
F
Lung fibrosis
Amphiregulin
BAFF
CD93
CCL2
MMP9
CCL6
IL1b
Gas 6
VCAM1
MCSF
IL4
IL2
FGF21
FiNav
DexNav
G
Liver fibrosis
CCL6
CCL5
CCL2
MMP9
MMP2
CXCL10
VCAM1
IL1b
Gas6
HGF
FGF-21
IFNy
IL2
IL7
EGF
GCSF
FidBET6
DexdBET6
H
Lung fibrosis
Amphiregulin
BAFF
CCL2
CCL5
MMP9
MMP2
IL5
VCAM1
IL1b
Gas 6
IL6
IL2
FGF21
IL4
FidBET6
DexdBET6

Figure S10: Impact of SMNPs on immune cells and cytokines in fibrotic, immunosuppressive niches. (Related to Figure 4)
(**A**) Representative immunohistochemistry analysis of $CD3^+$ T cells in fibrotic liver (top) and lungs treated with free navitoclax, free dBET6, DexNav, DexdBET6, FiNav and FidBET6 for 4 (liver) or 3 (lung) weeks. Scale bars, 75 µm. (**B**) Quantification from A in fibrotic livers (left) and lungs (right) (4 mice/group). (**C-D**) Representative mIF pictures of conventional DC 1 (cDC1), cDC2, tolerogenic DC (tolDC), and $CD40^+$ or $CD40^-$ activated DCs in murine fibrotic livers (C) and quantification in murine fibrotic livers and lungs treated for four weeks with DexNav, FiNav, DexdBET6, or FidBET6 nanoparticles (D). (**E-F**) Differential protein expression of cytokines from whole-tissue lysates of murine fibrotic livers (E) or lung (F) after 4 (liver) or 3 (lung) weeks of DexNav or FiNav nanoparticle treatment. (**G-H**) Differential protein expression of cytokines from whole-tissue lysates of murine fibrotic livers (G) or lung (H) after 4 (liver) or 3 (lung) weeks of DexdBET6 or FidBET6 nanoparticle treatment. Each dot represents one mouse (B). Data are presented as mean ± s.e.m. The one-way ANOVA with Sidak's multiple comparisons test was used for (B).

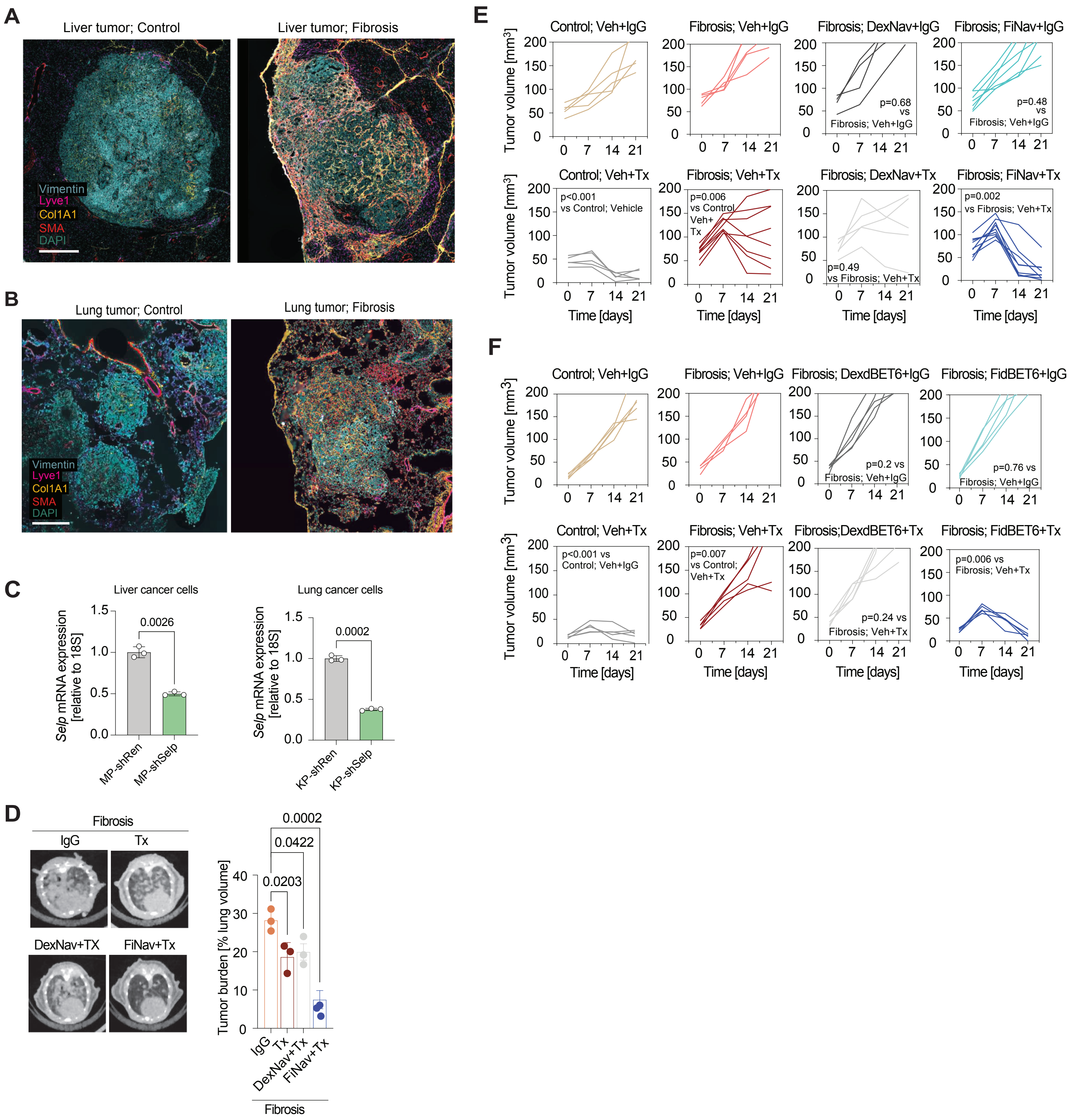

A
Liver tumor; Control
Liver tumor; Fibrosis
Vimentin
Lyve1
Col1A1
SMA
DAPI
B
Lung tumor; Control
Lung tumor; Fibrosis
Vimentin
Lyve1
Col1A1
SMA
DAPI
C
Liver cancer cells
Lung cancer cells
Selp mRNA expression [relative to 18S]
0.0026
0.0002
MP-shRen
MP-shSelp
KP-shRen
KP-shSelp
D
Fibrosis
IgG
Tx
DexNav+TX
FiNav+Tx
Tumor burden [% lung volume]
0.0002
0.0422
0.0203
IgG
Tx
DexNav+Tx
FiNav+Tx
Fibrosis
E
Control; Veh+IgG
Fibrosis; Veh+IgG
Fibrosis; DexNav+IgG
Fibrosis; FiNav+IgG
Tumor volume [mm³]
p=0.68 vs Fibrosis; Veh+IgG
p=0.48 vs Fibrosis; Veh+IgG
Control; Veh+Tx
Fibrosis; Veh+Tx
Fibrosis; DexNav+Tx
Fibrosis; FiNav+Tx
p<0.001 vs Control; Vehicle
p=0.006 vs Control Veh+ Tx
p=0.49 vs Fibrosis; Veh+Tx
p=0.002 vs Fibrosis; Veh+Tx
Time [days]
F
Control; Veh+IgG
Fibrosis; Veh+IgG
Fibrosis; DexdBET6+IgG
Fibrosis; FidBET6+IgG
Tumor volume [mm³]
p=0.2 vs Fibrosis; Veh+IgG
p=0.76 vs Fibrosis; Veh+IgG
Control; Veh+Tx
Fibrosis; Veh+Tx
Fibrosis;DexdBET6+Tx
Fibrosis; FidBET6+Tx
p<0.001 vs Control; Veh+IgG
p=0.007 vs Control; Veh+Tx
p=0.24 vs Fibrosis; Veh+Tx
p=0.006 vs Fibrosis; Veh+Tx
Time [days]

Figure S11: Impact of SMNPs on ICB responses in mice with fibrotic liver and lung tumors. (Related to Figure 5)

(**A**-**B**) mIF analysis of vimentin, Lyve1, Collagen type I (Col1A1), and smooth muscle actin (SMA) expression on fibrotic and non-fibrotic murine liver (A) and lung tumors (B). Scale bars, 250 µm. (**C**) Quantitative reverse transcription polymerase chain reaction (RT-PCR) analysis of *Selp* mRNA isolated from MP liver cancer cells (left) and KP lung cancer cells (right). 18S was used as the internal amplification control. (**D**) (Left) Representative image of microCT scan at d14 of murine fibrotic lung tumors treated with DexNav or FiNav nanoparticles in combination with lung first-line Tx. (Right) Quantification of microCT scans (n=3 mice/group). (**E**) Quantification of tumor volume change over time in murine fibrotic liver tumors treated with DexNav or FiNav nanoparticles in combination with liver first-line Tx (5-10 mice/group). (**F**) Quantification of tumor volume change over time in murine fibrotic liver tumors treated with DexdBET6 or FidBET6 nanoparticles in combination with liver first-line Tx (5-10 mice/group). Each dot represents one biological replicate (C), one mouse (D). Each line (E-F) represents one mouse. Data are presented as mean ± s.e.m. Unpaired t-test was used for (C). Statistical tests were the one-way ANOVA with Sidak's multiple comparisons test was used for (D), and the Mann-Whitney test or Welch's t-test at endpoint (21 days) was used for (E-F).

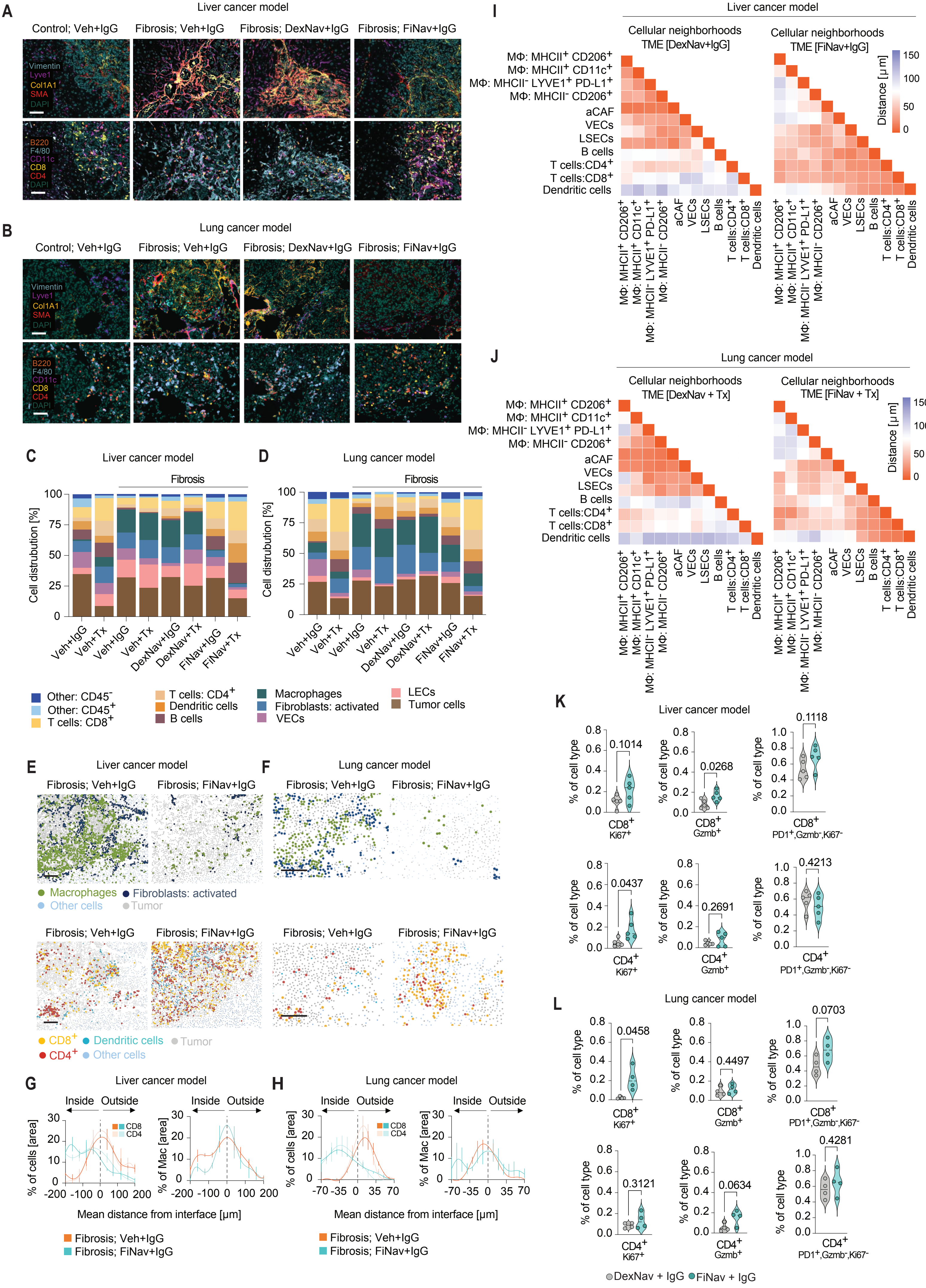
A
Liver cancer model
Control; Veh+IgG
Fibrosis; Veh+IgG
Fibrosis; DexNav+IgG
Fibrosis; FiNav+IgG
Vimentin
Lyve1
Col1A1
SMA
DAPI
B220
F4/80
CD11c
CD8
CD4
DAPI
B
Lung cancer model
Control; Veh+IgG
Fibrosis; Veh+IgG
Fibrosis; DexNav+IgG
Fibrosis; FiNav+IgG
C
Liver cancer model
Fibrosis
Cell distrubution [%]
Veh+IgG
Veh+Tx
Veh+IgG
Veh+Tx
DexNav+IgG
DexNav+Tx
FiNav+IgG
FiNav+Tx
D
Lung cancer model
Fibrosis
Cell distrubution [%]
Other: CD45-
Other: CD45+
T cells: CD8+
T cells: CD4+
Dendritic cells
B cells
Macrophages
Fibroblasts: activated
VECs
LECs
Tumor cells
E
Liver cancer model
Fibrosis; Veh+IgG
Fibrosis; FiNav+IgG
Macrophages
Fibroblasts: activated
Other cells
Tumor
CD8+
Dendritic cells
Tumor
CD4+
Other cells
F
Lung cancer model
Fibrosis; Veh+IgG
Fibrosis; FiNav+IgG
G
Liver cancer model
Inside
Outside
% of cells [area]
% of Mac [area]
CD8
CD4
Mean distance from interface [μm]
Fibrosis; Veh+IgG
Fibrosis; FiNav+IgG
H
Lung cancer model
Inside
Outside
% of cells [area]
% of Mac [area]
Mean distance from interface [μm]
Fibrosis; Veh+IgG
Fibrosis; FiNav+IgG
I
Liver cancer model
Cellular neighborhoods TME [DexNav+IgG]
Cellular neighborhoods TME [FiNav+IgG]
MΦ: MHCII+ CD206+
MΦ: MHCII+ CD11c+
MΦ: MHCII- LYVE1+ PD-L1+
MΦ: MHCII- CD206+
aCAF
VECs
LSECs
B cells
T cells:CD4+
T cells:CD8+
Dendritic cells
Distance [μm]
J
Lung cancer model
Cellular neighborhoods TME [DexNav + Tx]
Cellular neighborhoods TME [FiNav + Tx]
Distance [μm]
K
Liver cancer model
% of cell type
0.1014
0.0268
0.1118
0.0437
0.2691
0.4213
CD8+ Ki67+
CD8+ Gzmb+
CD8+ PD1+,Gzmb-,Ki67-
CD4+ Ki67+
CD4+ Gzmb+
CD4+ PD1+,Gzmb-,Ki67-
L
Lung cancer model
0.0458
0.4497
0.0703
0.3121
0.0634
0.4281
DexNav + IgG
FiNav + IgG

Figure S12: SMNP-mediated niche remodeling in fibrotic liver and lung tumors. (Related to Figure 5)

(**A-B**) mIF analysis of stromal (Vimentin, Lyve1, Col1a1, SMA) and immune (B220, F4/80, Cd11c, CD8, CD4) markers in murine fibrotic liver (A) or lung (B) tumors treated with DexNav or FiNav nanoparticles in combination with IgG controls. Scale bars, 100 µm. (**C-D**) Proportion of immune and stromal cells in murine fibrotic liver (C) or lung (D) tumors treated with DexNav or FiNav nanoparticles in combination with first-line Tx. (**E-F**) Representative images of mIF analysis mapping different immune (top) and stromal (bottom) populations in murine fibrotic liver (E) or lung (F) tumors treated with FiNav nanoparticles in combination with IgG controls. Scale bars, 100 µm. (**G-H**) Proportion of $CD8^+$, $CD4^+$ T cells (left) and macrophages (right) present inside or outside the tumor region in murine fibrotic liver (G) or lung (H) tumors treated with FiNav nanoparticles in combination with IgG controls. (**I-J**) Cellular neighborhood analysis measuring the distance between the different immune and stromal populations in murine fibrotic liver (I) or lung (J) tumors treated with DexNav or FiNav nanoparticles in combination with first-line Tx. (**K-L**) Quantification of active/cytotoxic/exhausted $CD8^+$ and $CD4^+$ T cells in murine fibrotic liver (K) or lung (L) tumors treated with DexNav or FiNav nanoparticles in combination with IgG controls (4-5 mice/group). Each line or dot represents one mouse. Data are presented as mean ± s.e.m. Statistical tests were Mann-Whitney test or Welch's t-test (K, L).

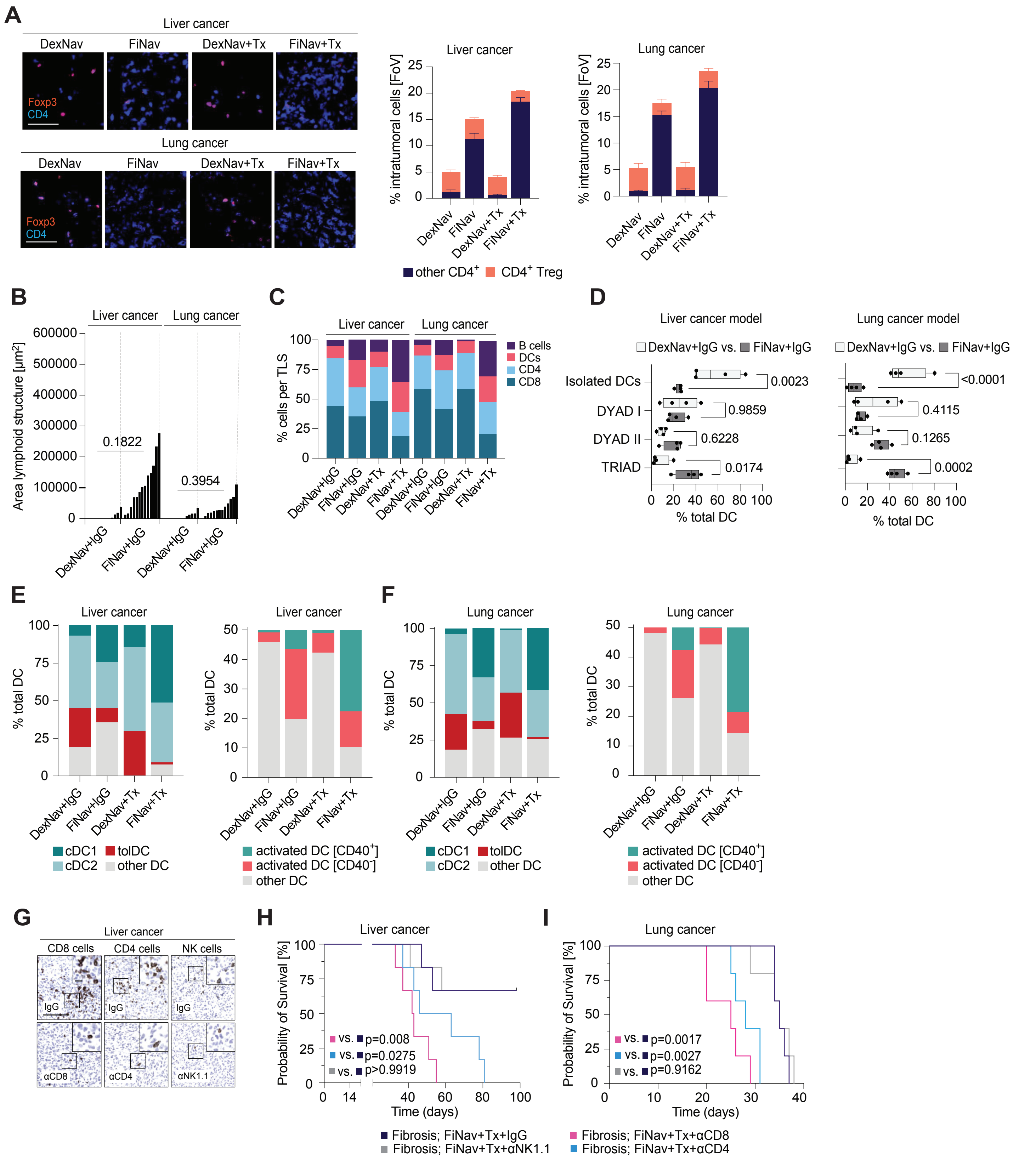
A
Liver cancer
DexNav
FiNav
DexNav+Tx
FiNav+Tx
Foxp3
CD4
Lung cancer
% intratumoral cells [FoV]
other CD4+
CD4+ Treg
B
Area lymphoid structure [µm²]
0.1822
0.3954
DexNav+IgG
FiNav+IgG
C
% cells per TLS
B cells
DCs
CD4
CD8
D
Liver cancer model
Lung cancer model
DexNav+IgG vs. FiNav+IgG
Isolated DCs
DYAD I
DYAD II
TRIAD
0.0023
0.9859
0.6228
0.0174
<0.0001
0.4115
0.1265
0.0002
% total DC
E
F
% total DC
cDC1
cDC2
tolDC
other DC
activated DC [CD40+]
activated DC [CD40-]
G
CD8 cells
CD4 cells
NK cells
IgG
αCD8
αCD4
αNK1.1
H
I
Probability of Survival [%]
Time (days)
p=0.008
p=0.0275
p>0.9919
p=0.0017
p=0.0027
p=0.9162
Fibrosis; FiNav+Tx+IgG
Fibrosis; FiNav+Tx+αNK1.1
Fibrosis; FiNav+Tx+αCD8
Fibrosis; FiNav+Tx+αCD4

Figure S13: Impact of SMNPs on lymphoid aggregates in the tumor microenvironment and dependence on $CD8^+$ T cells. (Related to Figure 5)

(**A**) Representative IF images showing $Foxp3^+$ regulatory T cells (Treg) and $CD4^+$ T changes during SMNP treatments. Quantification of $CD4^+$ and Treg in murine fibrotic liver (left) or lung (right) tumors treated with DexNav or FiNav nanoparticles in combination with first-line Tx. (**B**) Quantification of lymphoid aggregates in murine fibrotic liver and lung cancer treated with DexNav or FiNav nanoparticles in combination with IgG controls, related to figure 5M. (**C**) Proportions of B cells, dendritic cells, $CD8^+$ T cells, and $CD4^+$ T cells in lymphoid aggregates in murine fibrotic liver and lung cancer treated with DexNav or FiNav in combination with liver/lung first-line Tx (or IgG controls) (4 mice/group). (**D**) Quantification of isolated DCs, DC::CD4 dyads (DYAD I), DC:CD8 dyads (DYAD II), and DC::CD4::CD8 triads (TRIAD) in murine fibrotic liver (left) and lung (right) cancer treated with DexNav or FiNav in combination with IgG controls. (**E-F**) Quantification of (left) conventional DC 1 (cDC1), cDC2, and tolerogenic DC (tolDC) or (right) $CD40^+$ and $CD40^-$ activated DCs in murine fibrotic livers (E) and lung (F) cancer treated for four weeks with DexNav, FiNav, DexdBET6, or FidBET6. (**G**) Representative immunohistochemistry images of CD8, CD4, natural killer (NK) cells upon CD8, CD4, or NK cell depletion in murine fibrotic liver cancer. (**H-I**) Kaplan-Meier analysis comparing overall survival of mice with fibrotic liver (H) and lung (I) tumors treated with FiNav nanoparticles in combination with first-line Tx and depletion antibodies for either $CD8^+$ T cells, $CD4^+$ T cells, $NK1.1^+$ natural killer cells, or IgG controls. Each line or dot represents one mouse. Data are presented as mean ± s.e.m. Statistical tests were the Mann-Whitney test for (B), the one-way ANOVA with Sidak's multiple comparisons test for (D), and the log-rank Mantel-Cox test for (H, I).

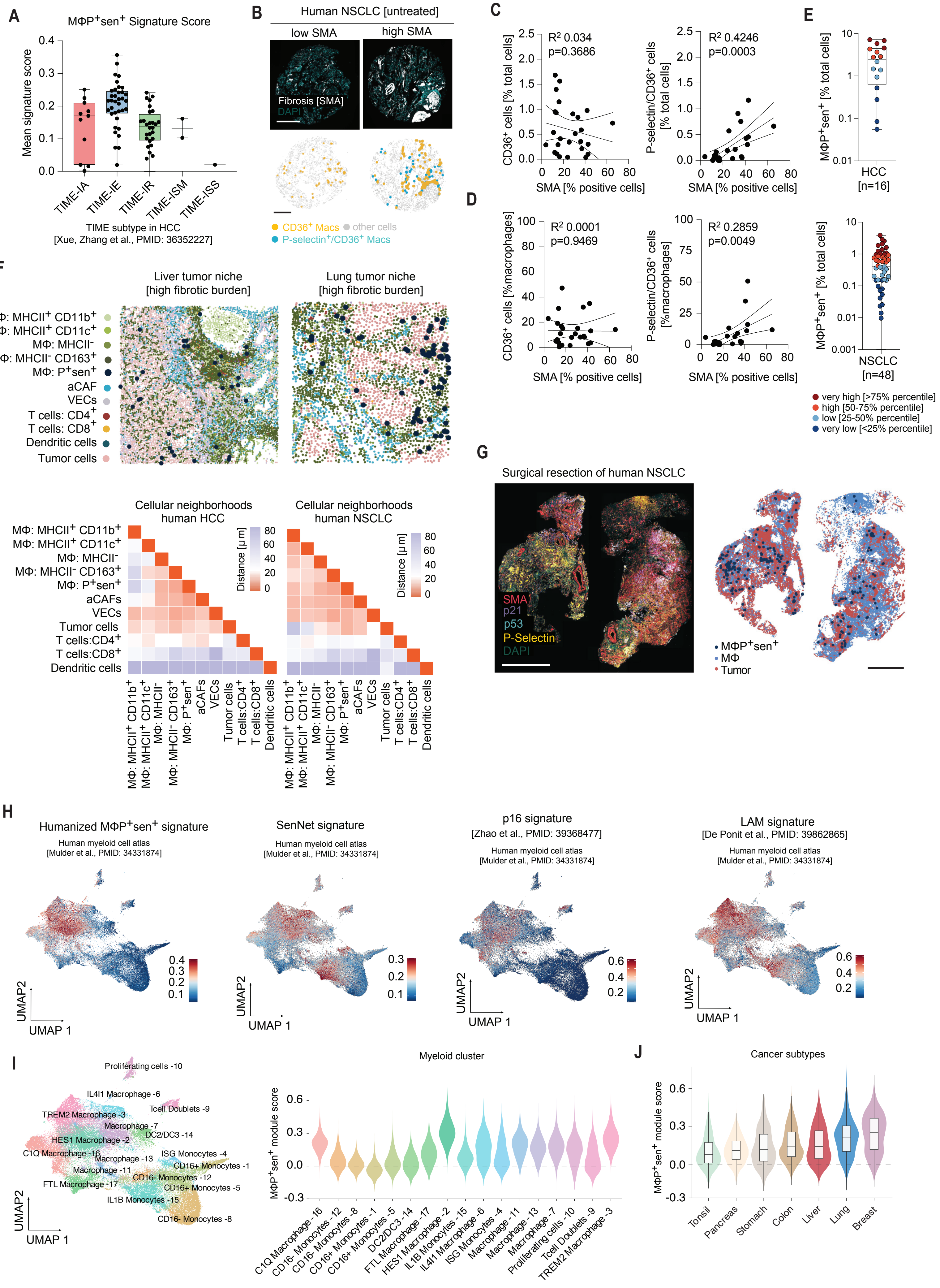

A
MΦP+sen+ Signature Score
Mean signature score
TIME-IA
TIME-IE
TIME-IR
TIME-ISM
TIME-ISS
TIME subtype in HCC
[Xue, Zhang et al., PMID: 36352227]
B
Human NSCLC [untreated]
low SMA
high SMA
Fibrosis [SMA]
DAPI
CD36+ Macs
other cells
P-selectin+/CD36+ Macs
C
R2 0.034
p=0.3686
CD36+ cells [% total cells]
SMA [% positive cells]
R2 0.4246
p=0.0003
P-selectin/CD36+ cells [% total cells]
SMA [% positive cells]
D
R2 0.0001
p=0.9469
CD36+ cells [%macrophages]
SMA [% positive cells]
R2 0.2859
p=0.0049
P-selectin/CD36+ cells [%macrophages]
SMA [% positive cells]
E
MΦP+sen+ [% total cells]
HCC
[n=16]
NSCLC
[n=48]
very high [>75% percentile]
high [50-75% percentile]
low [25-50% percentile]
very low [<25% percentile]
F
Liver tumor niche
[high fibrotic burden]
Lung tumor niche
[high fibrotic burden]
MΦ: MHCII+ CD11b+
MΦ: MHCII+ CD11c+
MΦ: MHCII-
MΦ: MHCII- CD163+
MΦ: P+sen+
aCAF
VECs
T cells: CD4+
T cells: CD8+
Dendritic cells
Tumor cells
Cellular neighborhoods
human HCC
Cellular neighborhoods
human NSCLC
Distance [μm]
aCAFs
G
Surgical resection of human NSCLC
SMA
p21
p53
P-Selectin
DAPI
MΦP+sen+
MΦ
Tumor
H
Humanized MΦP+sen+ signature
Human myeloid cell atlas
[Mulder et al., PMID: 34331874]
SenNet signature
Human myeloid cell atlas
[Mulder et al., PMID: 34331874]
p16 signature
[Zhao et al., PMID: 39368477]
Human myeloid cell atlas
[Mulder et al., PMID: 34331874]
LAM signature
[De Ponit et al., PMID: 39862865]
Human myeloid cell atlas
[Mulder et al., PMID: 34331874]
UMAP 1
UMAP2
I
Proliferating cells -10
IL4I1 Macrophage -6
Tcell Doublets -9
TREM2 Macrophage -3
Macrophage -7
DC2/DC3 -14
HES1 Macrophage -2
C1Q Macrophage -16
ISG Monocytes -4
Macrophage -13
CD16+ Monocytes -1
Macrophage -11
CD16- Monocytes -12
FTL Macrophage -17
CD16+ Monocytes -5
IL1B Monocytes -15
CD16- Monocytes -8
Myeloid cluster
MΦP+sen+ module score
J
Cancer subtypes
MΦP+sen+ module score
Tonsil
Pancreas
Stomach
Colon
Liver
Lung
Breast

Figure S14: Characterization of P$^+$sen$^+$ macrophages in human tumors. (Related to Figure 6)

(**A**) Signature score of P$^+$sen$^+$ macrophages across previously defined tumor immune microenvironment (TIME) classes of human HCC: Immune active (IA; n=11), Immune excluded (IE; n=36), Immune resident (IR, n=28), Immune suppressive myeloid (ISM; n=2) and Immune suppressive stromal (ISS; n=1). (**B**) Representative core image of mIF analysis mapping CD36$^+$ and P-selectin$^+$/CD36$^+$ macrophages in human NSCLC with a low or high fibrotic burden. Fibrotic burden has been quantified by a-smooth muscle actin (aSMA) expression. Scale bars, 250 μm. (**C**) Linear regression analyses between (left) CD36$^+$ cells (normalized to total cell number) and activated fibroblasts (aSMA) and (right) P-selectin$^+$CD36$^+$ cells (normalized to total cell number) and activated fibroblasts (aSMA) in human NSCLC tissues (26 patients) (**D**) Linear regression analyses between (left) CD36$^+$ cells (normalized to total macrophages) and activated fibroblasts (SMA) and (right) P-selectin$^+$CD36$^+$ cells (normalized to total macrophages) and activated fibroblasts (SMA) in human NSCLC tissues (26 patients). (**E**) IF quantification of P$^+$sen$^+$ macrophages in human HCC (top) and NSCLC (bottom) related to Fig. 6G-H. (**F**) (Top) Representative image of mIF analysis mapping different immune and stromal populations in human liver (left) and lung (right) tumor niche with high fibrotic burden (determined by aSMA level). (Bottom) Cellular neighborhood analysis measuring the distance between the different immune and stromal populations in human HCC (left) or NSCLC (right) tumors (analyzed in high fibrotic HCC (n=3)/NSCLC(n=3) samples). Scale bars, 150 μm. (**G**) Representative image of multiplex analysis mapping stromal, tumor, and macrophage populations in surgically resected human NSCLC tumor. Scale bars, 500 μm. (**H**) Humanized P$^+$sen$^+$ macrophage, SenNet, p16, and LAM signature in cross-tissue scRNA datasets of human myeloid cells in health and disease. (**I**) (Left) Cell type annotation and clustering as annotated by Mulder et al. (PMID: 34331874) and (right) P$^+$sen$^+$ macrophage signature score in each cluster. (**J**) P$^+$sen$^+$ macrophage signature score across cancer types. Each dot represents one patient (A, C-E). Data are presented as mean ± s.e.m. Simple linear regression was used for (C-D).

Supplementary table summary:

Table S1: Antibody List (A list of all antibodies used in mIF/IF/IHC).

Table S2: Cell type annotation (Marker combinations used to define cell types in mIF).

Table S3: scRNA-seq sample IDs (List including sample names and numbers of cells analyzed in each scRNA seq sample).

Table S4: Differentially expressed genes (DEG) in liver P^+Sen^+ macrophages (DEG list of P^+Sen^+ macrophages vs P^-Sen^- macrophages used for the P^+Sen^+ macrophage signature).

Table S5: Differentially expressed genes (DEG) in liver P^+Sen^+ macrophages (DEG list of P^+Sen^+ macrophages vs P^-Sen^- macrophages used for the P^+Sen^+ macrophage signature).

Table S6: Common P^+Sen^+ macrophages signature (list of shared DEGs between P^+Sen^+ macrophages in the lung and liver above 1LFC).
